# The genomes of *Crithidia bombi* and *C*. *expoeki*, common parasites of bumblebees

**DOI:** 10.1371/journal.pone.0189738

**Published:** 2018-01-05

**Authors:** Paul Schmid-Hempel, Markus Aebi, Seth Barribeau, Toshihiko Kitajima, Louis du Plessis, Regula Schmid-Hempel, Stefan Zoller

**Affiliations:** 1 Institute of Integrative Biology (IBZ), ETH Zurich, Zürich, Switzerland; 2 Institute of Microbiology, ETH Zurich, Zürich, Switzerland; 3 Genetic Diversity Centre (GDC), ETH Zurich, Zürich, Switzerland; University of Ostrava, CZECH REPUBLIC

## Abstract

Trypanosomatids (Trypanosomatidae, Kinetoplastida) are flagellated protozoa containing many parasites of medical or agricultural importance. Among those, *Crithidia bombi* and *C*. *expoeki*, are common parasites in bumble bees around the world, and phylogenetically close to *Leishmania* and *Leptomonas*. They have a simple and direct life cycle with one host, and partially castrate the founding queens greatly reducing their fitness. Here, we report the nuclear genome sequences of one clone of each species, extracted from a field-collected infection. Using a combination of Roche 454 FLX Titanium, Pacific Biosciences PacBio RS, and Illumina GA2 instruments for *C*. *bombi*, and PacBio for *C*. *expoeki*, we could produce high-quality and well resolved sequences. We find that these genomes are around 32 and 34 MB, with 7,808 and 7,851 annotated genes for *C*. *bombi* and *C*. *expoeki*, respectively—which is somewhat less than reported from other trypanosomatids, with few introns, and organized in polycistronic units. A large fraction of genes received plausible functional support in comparison primarily with *Leishmania* and *Trypanosoma*. Comparing the annotated genes of the two species with those of six other trypanosomatids (*C*. *fasciculata*, *L*. *pyrrhocoris*, *L*. *seymouri*, *B*. *ayalai*, *L*. *major*, and *T*. *brucei*) shows similar gene repertoires and many orthologs. Similar to other trypanosomatids, we also find signs of concerted evolution in genes putatively involved in the interaction with the host, a high degree of synteny between *C*. *bombi* and *C*. *expoeki*, and considerable overlap with several other species in the set. A total of 86 orthologous gene groups show signatures of positive selection in the branch leading to the two *Crithidia* under study, mostly of unknown function. As an example, we examined the initiating glycosylation pathway of surface components in *C*. *bombi*, finding it deviates from most other eukaryotes and also from other kinetoplastids, which may indicate rapid evolution in the extracellular matrix that is involved in interactions with the host. Bumble bees are important pollinators and *Crithidia*-infections are suspected to cause substantial selection pressure on their host populations. These newly sequenced genomes provide tools that should help better understand host-parasite interactions in these pollinator pathogens.

## Introduction

The Order Trypanosomatida (Class Kinetoplastea) are a diverse group of flagellated protozoans with many species of medical or agricultural importance [1–3]. Perhaps the best-known representatives are within the family Trypanosomatidae (the trypanosomatids), such as the "African Trypanosomes", like *Trypanosoma brucei* (causing human sleeping sickness), *T*. *vivax*, or the New World *T*. *cruzi*, the agent of Chagas disease [4]. In addition to these well-known examples, a range of other species are significant parasites of various organisms. For instance, *Leishmania* (such as *L*. *major*) cause a spectrum of human diseases collectively known as 'leishmaniasis' [5]. Most trypanosomatids, however, are pathogens of insects [6–9]; some infect plants (e.g. *Phytomonas*, [10]).

Here, we report on the nuclear genomic sequences of two species from the genus *Crithidia*, *C*. *bombi* Lipa & Triggiani 1988 [11] and *C*. *expoeki* Schmid-Hempel & Tognazzo 2010 [12], which are important and common parasites of bumblebees, *Bombus* spp. [13]. Together, the genus *Crithidia* as defined by the type species *C*. *fasciculata* ([14]) belongs to the newly proposed subfamily Leishmaniinae within the Trypanosomatidae [15,16]. The two taxa studied here are phylogenetically somewhat more distant to *C*. *fasciculata*, but close to the honeybee-infecting *C*. *mellificae*, at some distance from the honeybee parasite, *Lotmaria passim* [17], whilst also in close proximity of *Leptomonas* (e.g. *L*. *pyrrhocoris*, *L*. *seymouri*) [9,18–20] and *Crithidia thermophila* (formerly *C*. *luciliae thermophila)* ([14]). Bumblebees, on the other hand, are social insects that establish annual colonies of moderate size (up to a few hundred workers). As the colony cycle comes to an end, only the mated and inseminated daughter queens hibernate and establish their own colonies the next year. All other colony members, including the mother queen, perish before onset of winter. Ecologically, bumblebees are key pollinators in temperate and cool climates [21] and their services are of high economic value [22,23]. Currently, bumblebees are declining in abundance and diversity in many parts of the world [24–28], and parasites have been implicated in these losses [28,29]. In fact, *C*. *bombi* is known to castrate its host and to strongly reduce the fitness of founding queens in spring to a substantial degree [30]; this may also be the cause of the rapid and spectacular decline of the native *B*. *dahlbomii* in South America [28].

Many trypanosomatids are dixenous [18,31], that is, have two hosts, and depend on an insect vector for transmission. *Trypanosoma brucei*, for example, is vectored by tsetse flies (*Glossina*) and *Leishmania* uses sandflies (Phlebotominae). These parasites must therefore deal with the defence and immune systems of at least two groups of very different organisms. By contrast, *C*. *bombi* and *C*. *expoeki* have a life cycle with only one host (i.e., are monoxenous [8]) and are thus directly transmitted without the need of a vector. Within the nest of its bumblebee host, *Crithidia* is transmitted via infective cells on contaminated surfaces or by shared food; and between colonies via flowers that have previously been visited by other infected bees [32]. Also, the larvae within a colony can serve as a reservoir from which *C*. *bombi* can be transmitted further [33]. The infective dose is very low. Only a few dozens cells are necessary to establish infection, where, in the hind gut of the host bee, it reaches peak intensities after about a week [34]. As *Crithidia* cannot survive outside a living host for long [35], these parasites must not only find ways to rapidly infect individual hosts but also to persist in a host colony and to be passed on to the colony's daughter queens for overwintering, which is the only way to reach the next year's hosts [34]. Whilst residing in the host, *C*. *bombi* exhibits remarkably high genetic exchange among co-infecting strains [36]—far beyond what has been described in other trypanosomatids, such as *T*. *brucei* [37–39], *T*. *cruzi* [40], *L*. *major* [41], or *Crithidia fasciculata* [42].

Previous genomic studies of trypanosomatids have largely focussed on the dixenous taxa, such as *Trypanosoma* [43,44] and *Leishmania* [45,46]. Only more recently monoxenous representatives were added, e.g. *Lotmaria passim* (formerly, *Crithidia mellificae*) [17,47], or *Leptomonas pyrrhochoris* [19]. Collectively, these studies underline that the genomic organization of trypanosomatids is unusual in several respects [48]. For example, the characteristic, single kinetoplast, located at the base of the flagellar pocket, contains the kinetoplast DNA (kDNA), which is organized as a set of circular DNAs. The so-called maxi-circles (typically, a few dozen) contain fairly conserved regions coding for functions similar to mitochondrial genes in higher organisms (e.g. in *C*. *fasciculata*, maxi-circles are 38 kb in size), whereas the numerous (typically, many thousands), genetically heterogeneous mini-circles (2.5 kb in size) carry sequences that are involved in RNA-editing [49–52]. Moreover, genomic sequences are typically organized in polycistronic clusters (that is, the same mRNA is coding for different genes), the gene-coding regions generally lack introns, and post-transcriptional mechanisms rather than changes in transcription rate are used to regulate gene expression [53–57], whereby gene duplication is common to increase expression levels.

## Results

### Sequencing and assembly of the genome

The final genome assembly size of *Crithidia bombi* is 31.66 Mb and is well resolved with 206 scaffolds (Table 1); the GC-content is 55.8%. The scaffolds contain a total of 585 contigs with a N50-contig size of 124.6 kb. The average scaffold and contig length is 155.5 kb and 54.1 kb, respectively; scaffold N50 is 855 kb long. Based on the assembly size and the total amount of cleaned sequencing reads, the average sequencing coverage is estimated at 243-times. The CEGMA-analysis on the final assembly resulted in 179 complete (72.2%) and an additional 14 (5.6%) partial matches to core eukaryotic genes.

**Table 1:** Genome assembly statistics.

	*C*. *bombi* ^1^		*C*. *expoeki* ^2^
	Contigs	Scaffolds	Contigs
Assembly size (Mb)	31.66	31.66	34.08
Number ^3^^)^	585	206	222
Number > 1 kb	568	206	222
N50	124'651	855'437	592’188
Average length (bp)	54'120	155'526	153’504
Maximum length (bp)	536'807	2'546'452	3’079’598
GC content (%)	55.8	< same	54.4
GC content, coding (%)	61.3		59.9
Number of predicted genes (bp) ^4^^)^	7,808		7,851
Gene median length (nt)	1,352		1,352
Total coding sequence (nt)	14'130'019 (44.6%)		15'580'666 (45.7%)
Estimated coverage	243-times		62-times

^1)^ Assembly metrics according to the Functional Genomic Centre Zurich (FGCZ)

^2)^ for *C*. *expoeki*: no scaffolds, only contigs with no gaps.

^3)^ for contigs: scaffolds split at regions with at least 3 N.

^4)^ from: crithidia-bombi.GDC.2013.v1.all.maker.proteins.fasta; crithidia-expoeki.all.maker.proteins.fasta

Being the second species to be sequenced, and with the genome of *C*. *bombi* already at hand, we only used PacBio for *C*. *expoeki*, which lead to a lower, but still sufficient coverage (estimated at 62-times; Table 1). We thus managed to establish a high-quality genome of *Crithidia expoeki* with 222 contigs of 34.08 Mb (Table 1); the GC-content is 54.4%. The contigs do not contain any gaps, have an average length of 153.5 kb and a N50 size of 592.2 kb. The longest contig has a size of 3,079,598 bp. The CEGMA-analysis resulted in 184 complete (74.2%) and an additional 11 (4.4%) partial matches to core eukaryotic genes.

To check the completeness of the assembly, we also ran a BUSCO analysis [58]. For *C*. *bombi*, this showed 140 complete and single-copy, 1 fragmented and 74 missing BUSCO orthologs. For *C*. *expoeki* we found 148 complete and single-copy, 12 complete and duplicated, and 67 missing BUSCO orthologs. This compares well to the *L*. *major* values of 175 complete and single-copy, 10 complete and duplicated, and 40 missing BUSCO orthologs.

### Gene prediction and annotation

#### Crithidia bombi

The MAKER2-pipeline predicted 7,808 genes, roughly 90% of those without introns (S1 Table). The annotated genes for *C*. *bombi* are listed in S1 File (Crithidia-bombi.GDC.2013.v1.gff). In *C*. *bombi* we detected 213 polycistronic gene clusters with at least two genes. On average the gene clusters have 36 genes and are 142 kb long. Fifty-four clusters contain more than 50 genes, 14 clusters more than one hundred genes, and the largest cluster contains 214 genes. Functional annotation with Blast2GO resulted in 4,178 fully annotated protein sequences with InterPro, gene ontology (GO), and BLAST results. A further 408 sequences received GO and BLAST results, while 2,850 sequences only received BLAST hits. 372 protein sequences did not obtain any annotation at all. Of all the 7,436 top BLAST hits, 7,086 were to *Leishmania* species, 137 were to *Trypanosoma* species, and a further 106 to other published *Crithidia* species (excluding *C*. *expoeki)*. The species with the most top BLAST hits in NCBI was *Leishmania braziliensis* with 1,750 hits. The reciprocal best BLAST hit analysis against the *L*. *major* proteins resulted in 6,880 hits, representing 88.1% of all predicted proteins. Of these, 6,782 had a BLAST E-value less than 1 x 10^−20^ in both directions.

#### Crithidia expoeki

**Fig 1:**
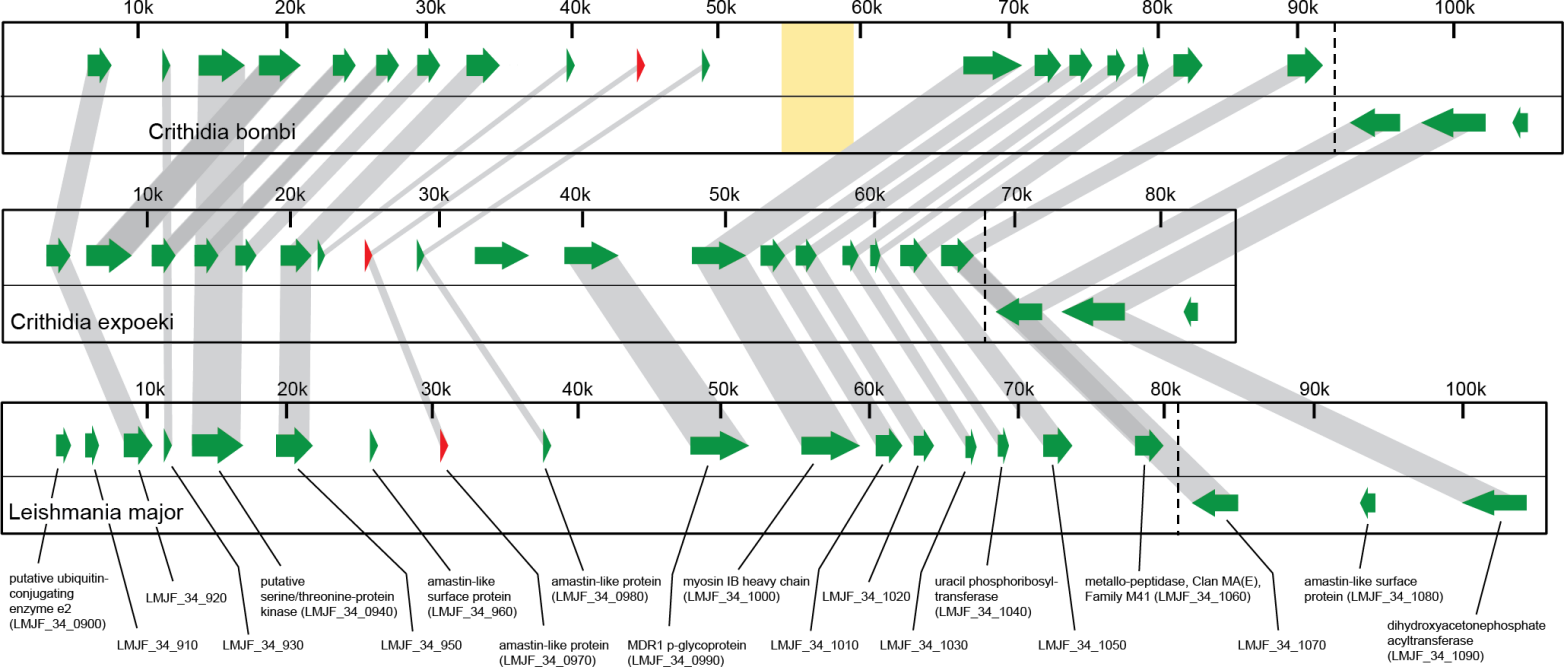
Genome organisation. Here we show sections of the genomes (kilobases, kb) of *C*. *bombi* and *C*. *expoeki* (top two panels; (scaffold3_*4* and scf7180000000921, respectively) and the syntenic region in *L*. *major* (bottom panel) as an example of overall synteny among these genomes. Green arrows are gene sequences coding for proteins, as based on annotations in *L*. *major* and as indicated at the bottom. Reversed (left-facing) arrows indicate polycistronic regions. Note that, in this example, no introns are present. The red arrow refers to the amastin-like protein (*LmjF*.*34*.*0970* in *L*. *major*), which is an ortholog to gene Ce.1.39770 (*C*. *expoeki*) and Cb.1.06720 (*C*. *bombi*). Two further amastin-like proteins are immediately up- and downstream from this location. The grey bars connect orthologs within the same orthologous group, as based on the OA analysis, and demonstrate a high degree of synteny among the three species. The yellow zone represents a gap in the *C*. *bombi* scaffold.

The MAKER2-pipeline predicted 7,851 genes, 82% of those without introns (S1 Table). The list of annotated genes for *C*. *expoeki* is in S2 File (Crithidia-expoeki.GDC.2016.v1.gff). In *C*. *expoeki* we counted 266 polycistronic gene clusters. On average the clusters have 29 genes and are 114 kb long. 51 clusters contain more than fifty genes, 11 clusters more than one hundred genes, and the largest contains 224 genes. While no systematic analysis of conservation (synteny) of polycistronic gene clusters between *C*. *bombi* and *C*. *expoeki* was conducted, manual inspection of a few clusters showed a rather high conservation among the two species. An example is shown in Fig 1.

Functional annotation of *C*. *expoeki* sequences with Blast2GO resulted in 4,973 fully annotated protein sequences with InterPro, gene ontology (GO), and BLAST results. A further 695 sequences received GO and BLAST results, 1,901sequences only received BLAST hits. A total of 282 protein sequences did not obtain any annotation at all. Of all the 7,566 top BLAST hits in NCBI, 6,701 were to *Leishmania* species, 81 were to *Trypanosoma* species, and a further 299 to other published *Crithidia* species (excluding *C*. *bombi*). The species with the most top BLAST hits was *Leishmania mexicana* with 1,455 hits. The reciprocal best BLAST hit analysis against the *Leishmania major* proteins resulted in 6,179 hits, representing 78.7% of all predicted proteins. Of these, 5,837 had a BLAST E-value less than 1 x 10^−20^ in both directions.

**Fig 2:**
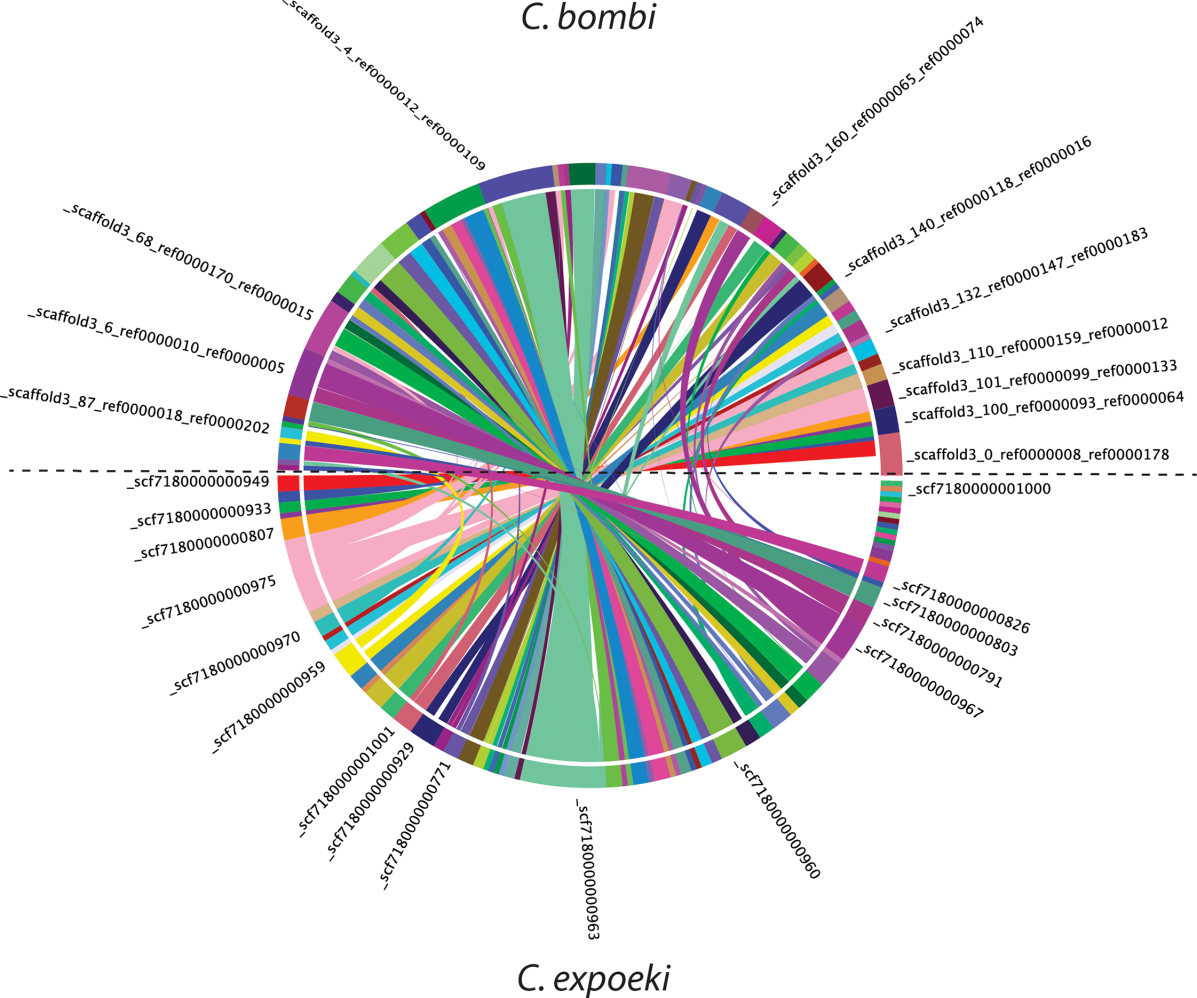
Synteny. Synteny graph between *C*. *bombi* and *C*. *expoeki* genomes generated with SyMap 4.2 [59,60]. The plot shows all syntenic blocks between the scaffolds of *C*. *expoeki* (bottom half of the circle) mapping to scaffolds of *C*. *bombi* (upper half of the circle). Each coloured block indicates a scaffold of the respective genome. Syntenic blocks are linked with lines in the colour of the *C*. *expoeki* scaffolds. For illustrative purposes, a few scaffolds (as named in this study) are indicated at their approximate position in the circle.

Thus, the two genomes had very similar number of predicted genes with similar support, and similar genomic architecture. For example, we found that a similar proportion of genes is encoded by a single exon, and a very similar distribution of exons per gene altogether. Furthermore, there is a high degree of synteny between the two species as shown in Fig 2. Quantitatively, the SyMap synteny analysis revealed 5,098 anchors in 99 syntenic blocks with conserved gene order among *C*. *bombi* and *C*. *expoeki* [59]. Of these conserved blocks, 18 are smaller than 100 kbp, 80 blocks are between 100 kbp and 1 Mbp, and one block is larger than one Mbp. The average block size in *C*. *bombi* is 299 kbp, the smallest block is 32 kbp, the largest 1.596 Mbp. Average block size in *C*. *expoeki* is 291 kbp, the smallest block is 27 kbp, and the largest 1.559 Mbp (S2 Table). The analysis for *C*. *bombi* and the seven additional species revealed between 71 syntenic blocks (with *T*. *brucei*) and 280 blocks (with *L*. *seymouri*). The analysis with *C*. *expoeki* and the additional species revealed between 75 syntenic blocks (with *L*. *major*) and 260 blocks (with *L*. *seymouri*). Thus, not surprisingly, *C*. *bombi* and *C*. *expoeki* have lesser degrees of synteny with the other taxa in the set (S1 and S2 Figs), notably with *T*. *brucei*. As expected from the phylogenetic distances, a high degree of synteny is detected with *L*. *pyrrhocoris*. A similar degree of synteny is expected with *L*. *seymouri*, however, the analysis is hampered by the comparably fragmented draft assembly (e.g., N50 is only 70 kbp), resulting in the majority of syntenic blocks being shorter than 100 kbp. Surprisingly, the two species under study here show rather high degrees of synteny with the phylogenetically more distant *Blechomonas ayalai* (S1 and S2 Figs).

**Fig 3:**
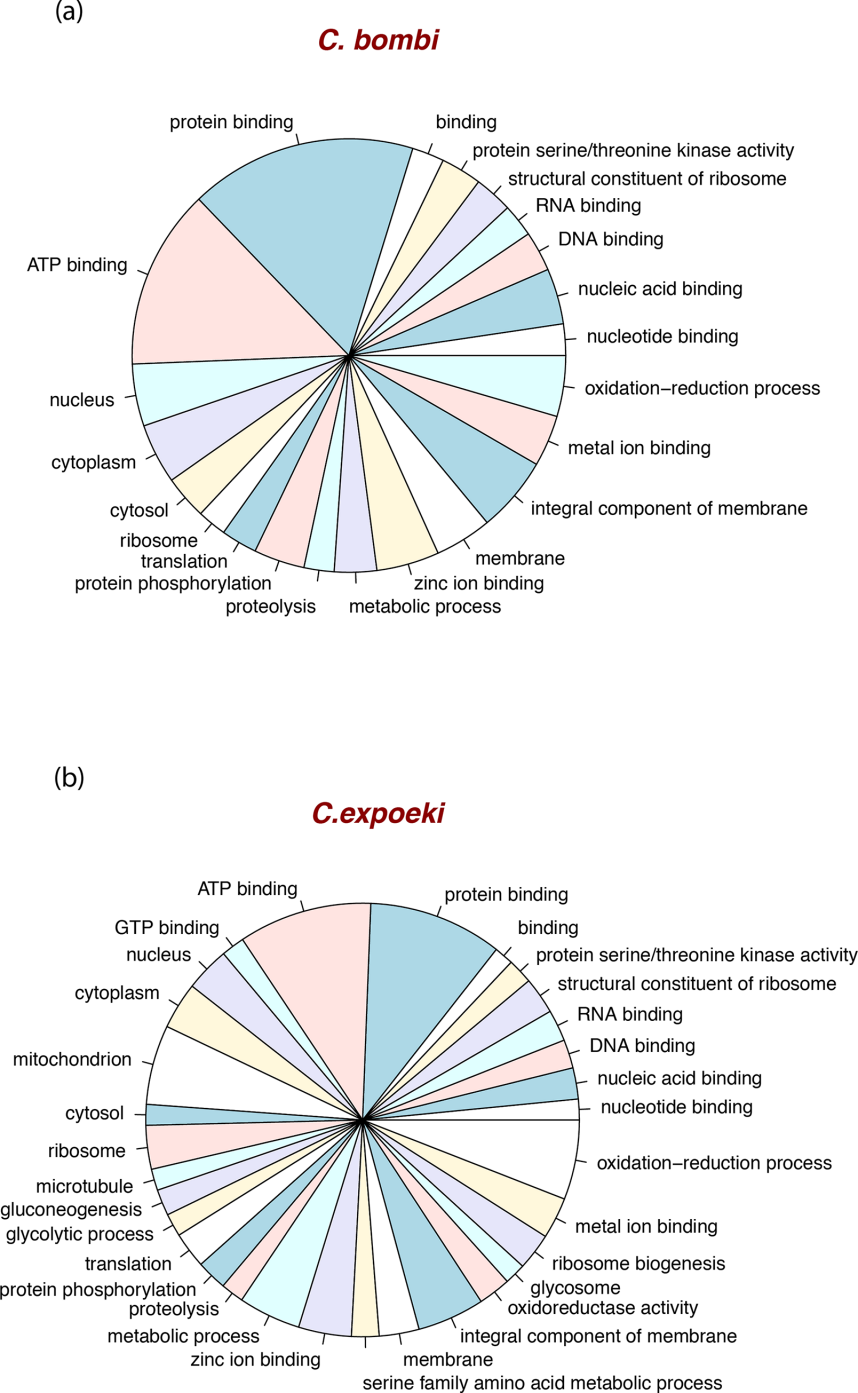
GO-categories in *Crithidia*. Pie diagrams of the GO-categories for the genes annotated here.(a) *C*. *bombi*, (b) *C*. *expoeki*. The analysis was done with Blast2GO [61]. Only terms with more than 100 members are shown here.

In addition, the distribution of GO-terms among the annotated proteins are, as expected, very similar in the two species under study (Fig 3). Differences were nevertheless visible, as there are more proteins in *C*. *bombi* that have been assigned to the protein- or ATP-binding categories, whereas *C*. *expoeki* seems richer in other categories, such as in serine family metabolic processes, glycolysis, or proteins associated with microtubules.

### Orthologs

The OMA browser identified orthologous sequences from the two genomes studied here relative to the set of the other trypanosomatid species as available in the TriTryp database (Table 2), and using the protein annotations for both *Crithidia* spp. from MAKER2 as described in the Materials and Methods and from the ENSEMBL Protist database. OMA also generated groups of orthologs that are shared between a minimum of two and up to all (eight) taxa in the comparison; these OMA groups were analysed further below.

**Table 2:** Number and types of orthologs.

Taxon	Genes ^1^	*C*. *bombi* ^2^	*C*. *expoeki* ^2^
*C*. *bombi*	7,808	-	5,448
this study			463
			8
			300
			(6,219)
*C*. *fasciculata*	9,619	6,548	5,728
TriTrypDB-33_CfasciculataCfCl		281	197
		8	346
		280	2,182
		(7,117)	(8,453)
*L*. *pyrrhocoris*	10,148	6,550	5,822
TriTrypDB-33_LpyrrhocorisH10		566	206
		14	353
		293	3,644
		(7,423)	(10,025)
*L*. *seymouri*	8,595	6,577	5,734
TriTrypDB-33_LseymouriATCC30220		8	4
		58	752
		30	46
		(6,673)	(6,536)
*B*.*ayalai*	8,126	4,917	4,386
TriTrypDB-33_BayalaiB08-376		23	16
		184	790
		43	66
		(5,167)	(5,258)
*L*. *major*	9,378	6,038	5,326
TriTrypDB-33_LmajorFriedlin		493	299
		35	423
		300	1,880
		(6,866)	(7,928)
*T*. *brucei*	11,703	3,683	3,348
TriTrypDB-33_TbruceiTREU927		1,017	780
		84	318
		540	1,932
		(5,324)	(6,378)

^1^ Gene count taken from TriTryp data base, except for *C*. *bombi* and *C*. *expoeki* (this study).

^2^ Entries are number of pairwise orthologs for either *C*. *bombi* or *C*. *expoeki* with the taxa listed on the left (respective data files indicated), as identified by OMA. Within each cell from top to bottom: number of orthologs of type '1:1', '1:many', 'many:1', 'many:many', and total orthologs (in parentheses).

**Fig 4:**
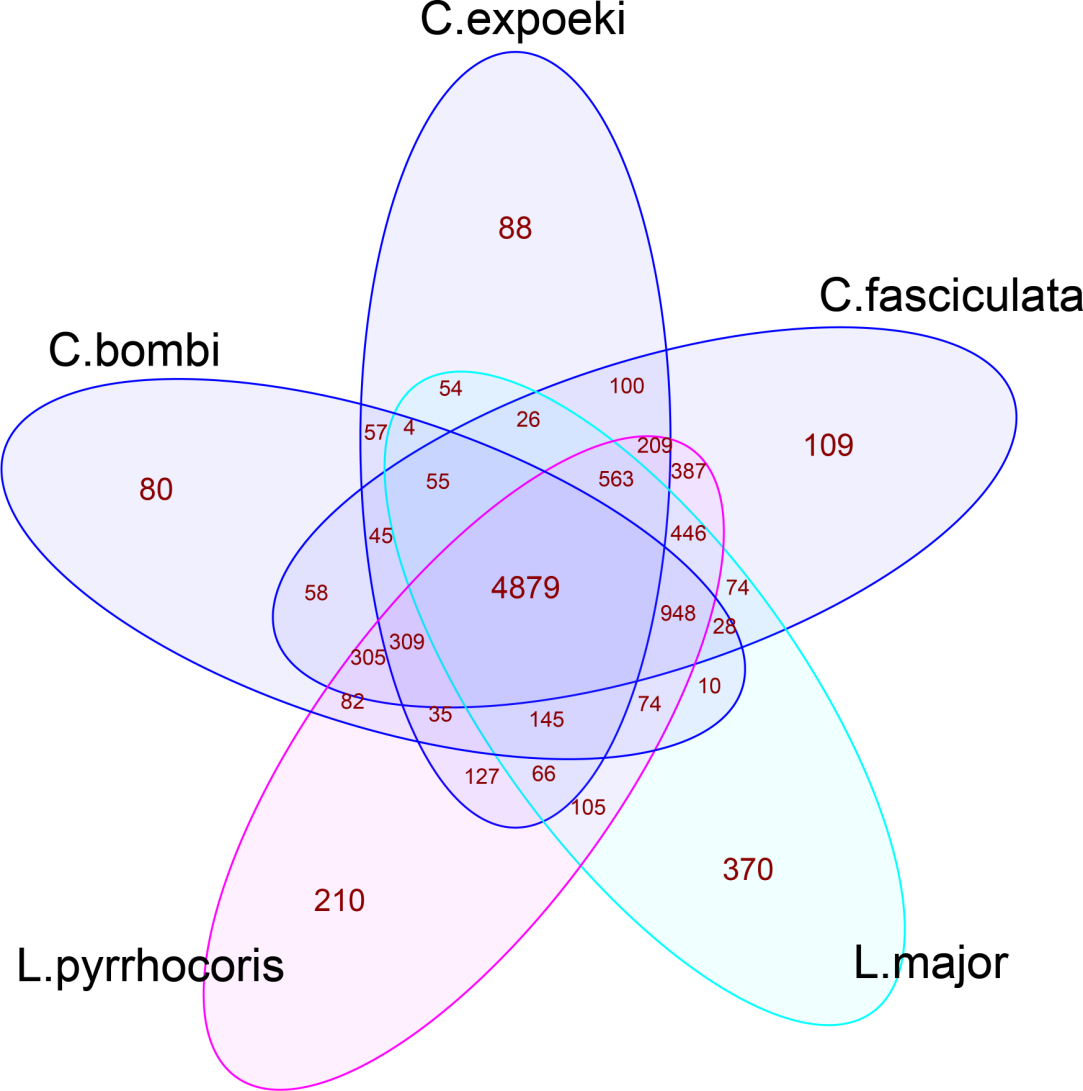
Venn diagram of orthologs. The Venn diagrams show the number of orthologs that are shared among a set of five species. Calculated with the OMA browser; matches of all types (1:1, many:1, 1:many, and many:many) are included.

Fig 4 shows the number of pairwise orthologs shared by the five species of prime interest. In all, the two *Crithidia*-species studied here have substantial overlap with *L*. *pyrrhocoris* (for *C*. *bombi*: a total of 6,777 orthologs overlapping, *C*. *expoeki*: 6,333), and marginally fewer with the type species, *C*. *fasciculata* (*C*. *bombi*: 6,599, *C*. *expoeki*: 6,186). There is a core of 4,879 orthologs that are shared among all taxa. *C*. *bombi* has a similar number of private proteins as *C*. *expoeki*, i.e. proteins that only are found in this taxon. The pairwise orthologs for the two *Crithidia*-species studied here with the other taxa are listed in S3 File.

Trypanosomatids share some of their physiology and ultrastructure, but, for example, surface molecules show lineage-specific elements [62,63]. These are of great interest, since the cell surfaces of parasitic protists interact with the host in many ways and thus can determine essential properties, such as infection success or parasite virulence. Because of the specialized mechanisms for polycistronic gene expression in trypanosomatids, genes coding for cell surfaces are organized in groups, the 'contingency gene families'. It is thought [63] that a process of concerted evolution, also observed in trypanosomatids, can lead to a loss of orthology in surface molecules because derived sequences, originating from gene duplication as paralogs, gradually replace the ancestral ones, such that the derived genes within one species are more closely related to one another than with the homologs in other taxa. Whilst the paralogs can shift to new genomic locations and assume new functions, the remaining orthologs occupy similar genomic positions throughout the clade.

Several surface gene families are of interest in this context, such as the Major Surface Proteases (MSPs) that are found across all trypanosomatids [63,64]. These show signatures of selection [65] and divergence among lines [64]. Typically, MSPs encode metalloproteases that counteract the host's immune defences; for example, in *Leishmania* MSPs block macrophage activity [66], among other effects [67]. MSP homologues and metalloprotease activities are known for several taxa in the narrow or more distant vicinity of *C*. *bombi* and *C*. *expoeki*, e.g. in *C*. *fasciculata*, *C*. *luciliae*, *C*. *deanei*, *C*. *guilhermei*, *Leptomonas seymouri*, *Bastocrithidia culicis* [67], or *Leptomonas pyrrhocoris* [19]. In the following cases, we extracted all orthologs that carried a particular annotation (such as 'gp63', or 'amastin') in the TriTryp database and reconstructed the phylogeny within the set of the eight species.

#### gp63

**Fig 5:**
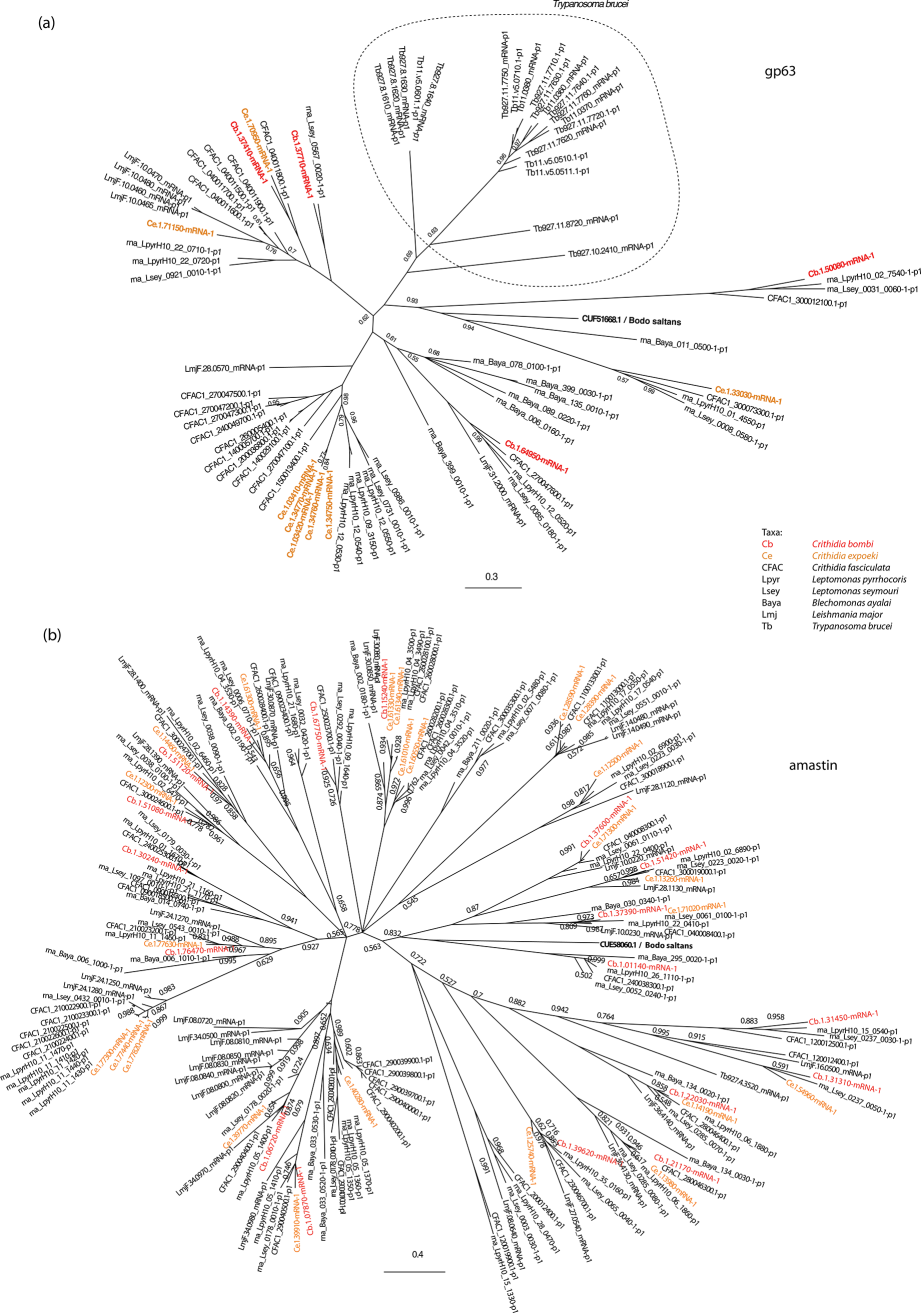
Phylogenetic relationships of orthologous proteins. Phylogenetic relationships of orthologous proteins in *C*. *bombi and C*. *exppoeki*, and as identified by OMA. Unrooted trees visualized with FigTree v.1.4.2 [70]; sequences from *C*. *bombi* (in red), and *C*. *expoeki* (in orange) shown in colour for clarity. Sequences of *Bodo saltans* (Kinetoplastida, Bodonidae; in bold black) represent a distant, outgroup kinetoplastid. Labels are as in TriTryp data base, and as named here for the two species under study. Branch values are posterior probabilities (PP), only values of PP < 1 shown here, all other cases have reported PP = 1. The horizontal bar is relative number of mutations per site. (a) *gp63*-like proteins. A total of 80 aligned, orthologous sequences were subjected to MrBayes (default settings, with 11 Mio generations and 25% burn-in fraction; convergence, S.D. of split frequencies < 0.004) to construct the consensus tree shown here. (b) *amastin-*like proteins. Tree from aligned, orthologous sequences submitted to MrBayes (default settings, 25% burn-in, with 12.6 Mio generations; convergence, S.D. of split frequencies = 0.01).

Among the MSPs, glycoprotein 63 (*gp63*) is one of the best studied [67]. It is involved in adhesion to host cells in *Leptomonas* [68]. We explored the relationships of *gp63* in the two *Crithidia* species studied here with those reported from the other trypanosomatids in our set of eight species. A total of four sequences for *C*. *bombi* and eight for *C*. *expoeki*, which met with the annotation 'gp63' in better studied taxa, were identified (see S3 Table). The other taxa in the set had a total of 69 sequences. Our phylogenetic analysis found all sequences of *T*. *brucei* clustered on a separate branch, whereas all other sequences intermingled with one another across the tree (Fig 5A). Except for one case (Ce.1.70950 pairing with Cb.1.37410), a given sequence for *C*. *bombi* was never closely associated with one from *C*. *expoeki*, and vice versa, whereas a conspicuous cluster of five sequences was found for *C*. *expoeki* only.

#### Amastins

These are transmembrane glycoproteins present in the surface of trypanosomatids and are expressed particularly in dixenous taxa when entering the mammalian host. In our set, we found a total of 190 sequences that met the criterion. Amastins have diversified in *Leishmania* (*L*. *pyrrhocoris* and *L*. *seymouri* together had 63 sequences), but orthologs are also found in the monoxenous species such as *Crithidia* [69], with *C*. *fasciculata* alone contributing 43 sequences in our study. Our annotation had identified 18 sequences in *C*. *bombi*, and 24 sequences in *C*. *expoeki*. The phylogenetic analysis (Fig 5B) showed that amastins of the two species are found in all parts of the tree and group with those of other taxa in a seemingly arbitrary way. With few exceptions (e.g. the clusters containing Ce.1.77300, or Ce.1.28590; Fig 5B), *C*. *bombi* and *C*. *expoeki* always have representatives each within the same neighbourhood (clusters defined by the longer branches followed by small radiations; Fig 5B), suggesting that diversification of amastins in the two species happened in parallel several times along the different branches. Also, amastins of the two species are often nearest to *Leptomonas* or *C*. *fasciculata* (Fig 5B).

#### *Tryparedoxin* and *RAD51*

These enzymes are unique for trypanosomatids and essential for their infection success, for example, qualifying as 'virulence factor' in *Leishmania* [71]. Their biological role is thought to be in the mitigation of host defences by oxidative stress [72,73], but this process may actually be independent of tryparedoxin, at least in *L*. *infantum* [74]. In our analysis, we find a total of 96 *tryparedoxin*-like sequences in the set of eight species, with 11 sequences in *C*. *bombi* and 14 in *C*. *expoeki*. The reconstructed phylogeny suggests a similar pattern as found in the amastins, i.e. the tryparedoxins of the two species are found at different places in the tree, and the clusters contain representatives of each (except Ce.1.65800 and Ce.1.65740) (S3A Fig)

*C*. *bombi* shows frequent genetic exchange when different genotypes co-infect the same host [36], and the recombination pattern is consistent with Mendelian segregation as also reported in other trypanosomatids [39]. *RAD51*, for example, is part of the recombination system that underlies the variable expression of surface molecules and which is based on an archive system as best described for the 'African Trypanosomes' [75,76]. In this process, the infecting parasite changes its antigenic surface by retrieving variants from the archive in a programmed way such as to escape the detection by the host's immune system [77–79]. In our study, OMA identified at total of 31 sequences carrying a 'RAD51'-annotation in the set of eight taxa, of which four each were assigned to *C*. *bombi* and *C*. *expoeki*, respectively. Again, the same pattern as above emerged, with the sequences of the two species found in pairs around the tree (S3B Fig).

### Signatures of selection

For this exploratory analysis, a total of *n* = 2,934 one-to-one orthology groups with entries for all eight species was available. Across the whole phylogeny (testing model M8 vs. M7 from PAML for strict trimming), a total of 350 orthology groups showed signs of significant selection and had some annotation information, whereas the remaining 119 groups had no annotation (based on annotations for *C*. *bombi*). In the example of strict trimming (S4 File), the most common meaningful annotations were 'dynein heavy chain' (*n* = 8 groups), 'ATP-dependent RNA helicase' (*n* = 6), 'protein kinase' (*n* = 4), or 'ABC transporter' (*n* = 15), whereas un-informative groups, such as ' hypothetical protein' (*n* = 32) and 'missing' (*n* = 158) were most frequent. We eventually found 380 orthologous groups showing evidence of positive selection (after Benjamini-Hochberg correction) for all trimming criteria; no trimming resulted in the most groups (*n* = 919), followed by 'relaxed' (*n* = 685) and 'strict' (*n* = 469) (S5 File, S4 Fig).

We also detected evidence of significant positive selection on the branch leading to *Crithidia* (BS model from PAML). A total of 86 groups tested significant for positive selection (after Benjamini-Hochberg correction) for all trimmings. The largest number of groups showed significance with no trimming (*n* = 522), followed by 'relaxed' (*n* = 316) and 'strict' (*n* = 91) (S6 File, S5 Fig). The significant groups in the BS-model contained very similar annotations as for the M8-model, as shown with the example of strict trimming (S4 File). Among the 91 significant groups, the most frequent categories were 'missing' (*n* = 33 groups) and 'hypothetical protein' (*n* = 14), followed by 'dynein heavy chain' (*n* = 3), and many others that appeared only once.

To test whether genes are evolving more rapidly in *Crithidia* than in the rest of our trypanosomatid tree, we compared the signature of selection from the M8-model that covers the whole phylogeny to the results from the branch-site (BS) model that calculates the selection only on the branch leading to *Crithidia*. Only groups that tested significant at *P* < 0.05 after a Benjamini-Hochberg correction, and that were significant in both models (M8 and BS) were included; a total of 23 groups met this criterion. Yet, the BS-model generated very high values of ω, which are biologically unlikely and may result when the proportion of sites assigned to ω > 1 is very small or when there is not enough information in the data to accurately infer the value of ω. Only one group (ID 261, annotated with 'dynein heavy chain') was within reasonable limits (i.e. in the range ω < 10).

### Metabolic pathways: Example of N-glycan

As an example of the metabolic pathways found in *Crithidia*, we checked the pathway that leads to N-glycans of *C*. *bombi*, which, in eukaryotes, have many functional roles. N-glycans are assembled in the endoplasmic reticulum, transferred to selected asparagine residues (N-X-S/T sequon) of polypeptides that enter the secretory pathway and are further modified in the Golgi. In this study, we could not fully resolve the structure of the N-glycans, but instead focus on the evolutionary analysis of critical enzymes in the cascade, especially on ALGs ('asparagine-linked glycosylation'). These are enzymes that catalyse the addition of sugars to conserved oligosaccharide precursors in the endoplasmic reticulum.

**Fig 6:**
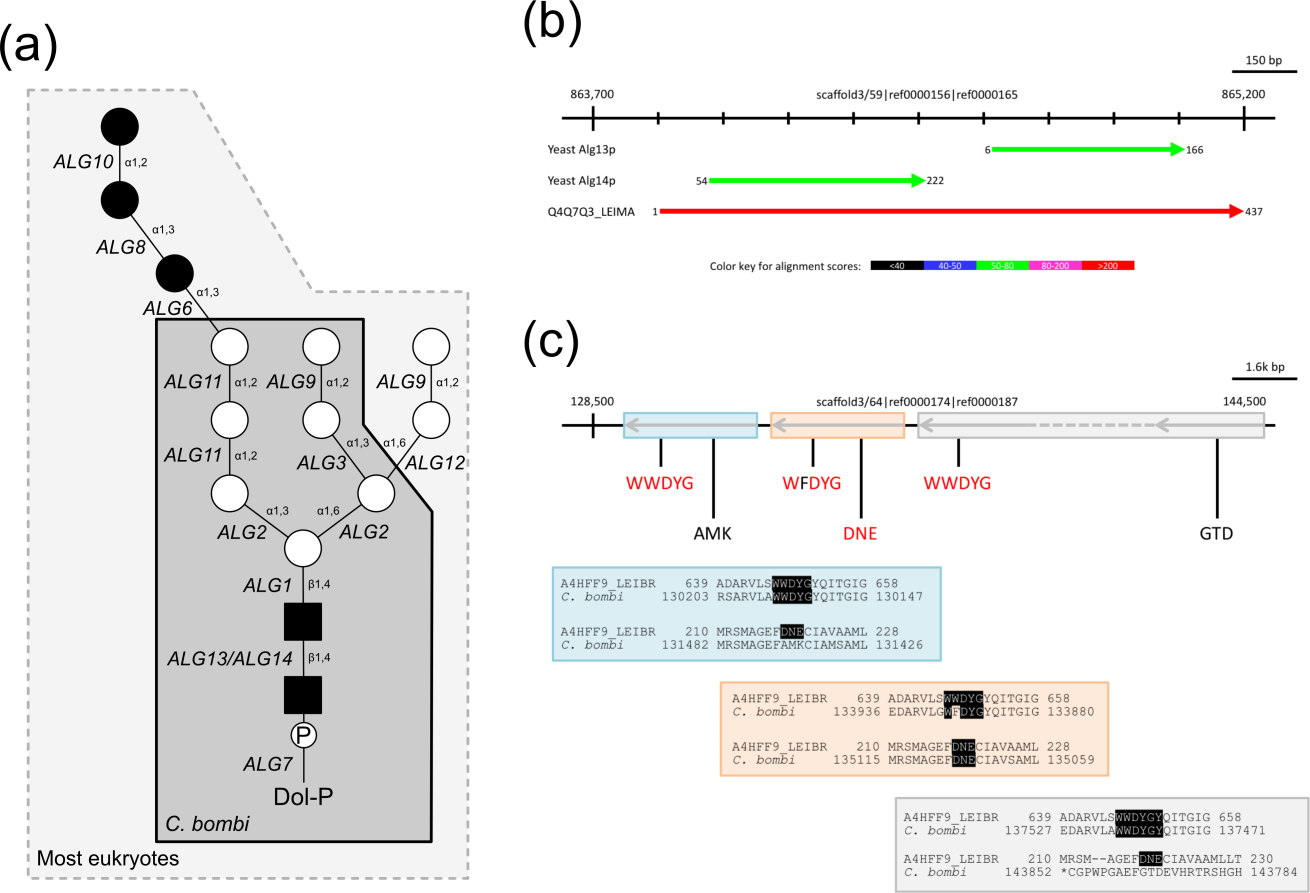
Putative N-glycan synthesis in *C*. *bombi*. (a) The complete N-glycan precursor synthesized in most eukaryotes (surrounded by dashed line) is composed of two *N*-acetylglucosamines (black squares), nine mannoses (white circles), and three glucoses (black circles). However, because *C*. *bombi* lacks *ALG6*, *ALG8*, *ALG10*, and *ALG12* genes, it was assumed that *C*. *bombi* synthesizes biantennary DolPP-GlcNAc2Man7 (grey box, surrounded by solid line). Genes encoding ALG glycosyltransferases responsible for the addition of each carbohydrate are shown in italics together with linkage information. (b) Alignment of yeast Alg13 and Alg14 to a scaffold in the *C*. *bombi* genome, showing that these two enzymes are encoded by a fused gene on this scaffold 3/59. **(c)** Alignments of *Leishmania braziliensis* STT3 to a scaffold in *C*. *bombi* (scaffold 3/64).

In our analysis, we blasted putative genes from *C*. *bombi* against known sequences from yeast in the data bases. We found that N-glycan synthesis in the endoplasmic reticulum must be different from other eukaryotes. As is the case for other parasitic kinetoplastids [80], *C*. *bombi* lacks genes involved in the glucosylation of LLOs (lipid-linked oligosaccharides) in the luminal side of the endoplasmic reticulum, that is, *ALG 5*,*6*,*8*,*10*. In addition, the absence of the *ALG12* locus (encoding a Dol-P-Man-dependent α-1,6 mannosyltransferase) suggests the transfer of a Man7GlcNAc2 oligosaccharides by oligosaccharyltransferase (OST; Fig 6, S6 Fig), which is similar to *Crithidia* and *Leishmania* but different from other trypanosomatids. *ALG13* and *ALG14* encode glycosyltransferase subunits, responsible for the second GlcNAc-addition (*N*-acetylglucosamine) to the LLO. The catalytic activity is with Alg13, but this is not activated unless when bound to Alg14—at least based on what is known from yeast [81]. In *C*. *bombi*, the linker sequence in the fused Alg13/Alg14 does not seem to take part in the folding of the active protein, although we cannot conclusively confirm this, as the protein structure could not be fully analysed. Alg13/Alg14 are encoded as a fused gene on a scaffold of the *C*. *bombi* genome (Fig 6B).

Furthermore, *C*. *bombi* does not encode any non-catalytic subunits of oligosaccharyltransferase (OST; Fig 6), but three paralogs of catalytic subunit Stt3 (S7 Fig) located on scaffold 3/64. One of them has a large insertion sequence and thus might be a vestigial gene. We cloned the remaining two paralogs (*CbSTT3A*, *CbSTT3B*) for functional tests. It appears from these tests that *CbSTT3A* but not *CbSTT3B* can functionally complement the mutant *stt3* from yeast (S8 Fig). *CbSTT3B* has a mutation in the DxE motif (corresponding to a DxD motif involved in binding a divalent cation [82,83]). It has been suggested that the ALG-glycosyltransferase set is secondarily lost [84]. Alternatively, the eukaryotic ALG pathway might have evolved by the addition of endoplasmic reticulum lumen-oriented glycosyltransferases [85]. Irrespective of the model applied, the systems in *Crithidia* and *Leishmania* were probably branching from a common ancestor that itself branched from *Trypanosoma*. Because *Leishmania* species encode three active *STT3* genes [86], the inactivation of *CbSTT3*s may have happened early as the lineage split from the common ancestor.

## Discussion

The biology of the genus *Crithidia* and, in particular, *C*. *bombi* has been the focus of a number of ecological, evolutionary studies over the last two decades. Taken together, these have explored and analysed the wide-spread occurrence of this parasite, e.g. [87–90], its effects on the host [30,91,92], the genetic structure of populations [36,88,89], the dynamics of multiple infections [93], or scrutinized the host genes and their expression when defending against this pathogen [94–96]. At the same time, the toolbox to study such questions had expanded over the years. For example, polymorphic microsatellites and SNPs are now available for *C*. *bombi* [36]. Both, *C*. *bombi* and *C*. *expoeki*, can be extracted from infected wild bees, and clones from single cells established, multiplied and maintained in pure culture [97]. The *Bombus-Crithidia* system is therefore very accessible for field work and laboratory studies.

Sequencing and annotation of the full genomes of *C*. *bombi* and *C*. *expoeki* now adds further important elements to the toolbox and adds a substantial amount of detailed information on these parasites. For *C*. *bombi*, the two-step assembly procedure resulted in an estimated genome of 31.66 Mb size, similar to the 34.08 Mb of *C*. *expoeki* (Table 1). These numbers are also similar to the estimated genome sizes of other kinetoplastids, such as *Leishmania major* Friedlin (32.8 Mb [45,48]), *Trypanosoma brucei* (25–26 Mb [43]), but somewhat smaller than *T*. *cruzi* (55 Mb [48]). Furthermore, our analysis suggests that both, *C*. *bombi* and *C*. *expoeki*, have around 7,800 protein-coding genes (Table 2). These numbers are quite comparable with estimates for *L*. *seymouri* (8,595 genes), and *B*. *ayalai* (8,126), but somewhat smaller than those in the other species of the set (*c*.*f*. Table 2). Some of this difference can be accounted for by the much more detailed study that some of the other species, such *L*. *major*, *T*. *brucei*, or *C*. *fasciculata*, have received. Alternatively, it could reflect evolutionary change that, for example, can lead to lineage-specific gene loss as a result of adaptation to parasitism and specific host groups (e.g. [9,46,80]. The average polycistronic gene cluster length of 142 kb in *C*. *bombi* and 114 kb in *C*. *expoeki* are slightly shorter than the sizes predicted for pol II clusters in *T*. *brucei* (153 kb) and *L*. *major* (180 kb) [98].

Taken together, we can see that the two genomes reported here are quite typical for the trypanosomatids, and the kinetoplastids more generally, in many of their aspects, such as genomic organisation (Figs 1 and 2) or the gene repertoire (Fig 3). Just like other trypanosomatid parasites [48,99], the two *Crithidia* species studied here also show polycistronic gene organisation and relatively few introns (S1 Table). At the same time, the phylogenetic reconstruction of gene trees shows a pattern consistent with concerted evolution, also similar to other trypanosomatids. The sequences of *C*. *bombi* and *C*. *expoeki* occur in 'pairs' (with 'pairs' sometimes meaning more than two sequences) within the same clusters and these 'pairs can be located in different parts of the tree (Fig 5, S3 Fig). In other words, these *C*. *bombi* and *C*. *expoeki* genes are each closely related to one another and to homologous sequences in the other trypanosomatids, but more distant from further, functionally similar genes (based on current annotations) within the same organism. An interesting slight deviation is visible for our reconstruction of *gp63* (Fig 5A) because the sequences from *T*. *brucei* are conspicuously separated from all others, whilst *C*. *expoeki* shows an expansion to five sequences and seems more separated from *C*. *bombi* than observed in the other genes studied.

Because of the unusually high rates of genetic exchange observed in *C*. *bombi* [36], genes associated with the recombination machinery are particularly interesting. As an example, *RAD51* is a conserved part of the recombination system that otherwise underlies the variable expression of surface molecules in the 'African Trypanosomes' [75,76]. We identified several orthologs of *RAD51* in the two *Crithidia* species studied here, and, again, indications of concerted evolution, which can lead to the observed pairing of sequences from *C*. *bombi* and *C*. *expoeki* in different parts of the tree (S3B Fig). The function of *RAD51* in the *Crithidia* species studied here, and whether their recombination system may be different, must remain unclear for the time being.

Our current analysis is still rather preliminary but for some genomic aspects, *C*. *bombi* and *C*. *expoeki* are closer to *Leptomonas* (especially, *L*. *pyrrhochoris*[19]) than, for example, to *C*. *fasciculata*—the 'type' species—or *C*. *mellificae* (*Lotmaria passim*)—a parasite of the honeybee [17]. On the other hand, *C*. *bombi* and *C*. *expoeki* are distinct in many aspects from the rest. For instance, a number of genes appear to be under positive selection in the branch leading to *Crithidia* (S6 File), many of those with unknown function, yet, 'dynein heavy chain' appearing prominently (S4 File). These proteins are typically associated with microtubules and involved in flagellar movement, e.g. [100]. Looking at the example of the initiating glycosylation pathway of surface components, we also found that the two *Crithidia* studied here deviate from most other eukaryotes and are also somewhat different from other kinetoplastids. For example, the two species lack various ALG glycosyltransferases, and all of them lack non-catalytic OST subunits that are typical for the eukaryotic pathway (Fig 6, S6–S8 Figs). The observed differences in canonical kinetoplastid N-glycosylation pathways could be the result of a rapid evolution of the glycan components of the extracellular matrix in kinetoplastids, possibly driven by a strong selection pressure exerted by the defence system of the host. Hence, we find many similarities to other trypanosomatids and a few differences. In this first study, however, there are no conspicuous, unique genomic features that could readily be associated with the simple, direct life cycle of the two species studied here and which would contrast with those genomic characteristics of species that have more than one hosts and/or a vector.

*C*. *bombi*, and very likely *C*. *expoeki* too, are important pathogens of bumblebees and arguably represent a considerable threat for the provision of pollination services by these bees. For example, infected spring queens loose nearly half of their reproductive success, thus exerting considerable selection on host populations [30]. These newly sequenced genomes represent another major step to better understanding these host-parasite interactions. Clearly, these genomes need to be analysed in more detail and, in particular, more functional tests are needed to develop a deeper insight into the genetic underpinnings of the infection and virulence processes.

## Materials and methods

### Origin of samples and DNA extraction

We isolated our reference strain of *C*. *bombi* from a spring queen of *Bombus terrestris* L., collected on April 10, 2008, in Northeastern Switzerland (site: 'Neunforn'). We separated an individual cell and grew it clonally according to the methods described in [97] (designated as clone #08.076 in our project archives). We then extracted genomic DNA with the Blood & Cell Culture DNA Midi Kit (Qiagen, cat. no. 13343) according to the smanufacturer's instructions. We similarly isolated and extracted genomic DNA for a strain of *C*. *expoeki* from a *B*. *lucorum* worker collected in the Jura mountains (site 'Röschenz', on June 9, 2008) (clone #BJ08.175).

### Genome sequencing

We sequenced the full genome of *Crithidia bombi* using a combination of sequencing runs on the Roche 454 FLX Titanium, Pacific Biosciences PacBio RS (starting in 2009; both at the Functional Genomics Center Zurich, FGCZ; http://www.fgcz.ch), and Illumina GA2 at GATC-Biotech (Konstanz, Germany). We used a total of 11 fragment libraries constructed for the 454-platform (9 paired-end libraries and 2 single-end libraries; S3 Table). In total, we generated 7,127,289 sequence reads with a mean length of 446 bp on this 454-platform. We produced one single-molecule real-time (SMRT) library for the PacBio platform according to the manufacturer's recommendations (Pacific Bioscience; but slightly modified, as we had to start with 10–20 μg, rather than 5 μg as suggested, sheared with g-tubes from Covaris, pn 520079), and then sequenced the library at the FGCZ on six SMRT cells and according to FGCZ's protocols. Our PacBio sequencing generated 270,958 sequences with mean length of 2,517 bp. For the Illumina, we constructed and sequenced four fragment libraries, producing 65,082,902 single-end reads of 76 bp length. Reads containing adapters were trimmed with cutadapt [101]. Quality-filtering and trimming was done with condetri.pl [102]. We used the Illumina reads to error-correct the PacBio reads with the pacBioToCA module of the WGS-Assembler version 7.

We sequenced the full genome of *Crithidia expoeki* with the Pacific Biosciences PacBio RS platform at the FGCZ. One SMRT library was constructed and sequenced on 9 SMRT cells, generating 381,293 sequences with a mean length of 7,181 bp; trimming was done within a local installation of the Pacific Bioscience SMRT portal version 2.3.0.

### Genome assembly

We assembled the *Crithidia bombi* genome in two steps. First, we assembled all Roche 454 sequence reads using the runAssembly command line interface of the 454 GS de novo assembler version 2.7 with default settings, except for minimum overlap length (set to 40 bp), minimum overlap identity (set to 95%) and minimum contig length (set to 100 bp). The resulting assembly contained 265 scaffolds, a scaffold N50 of 658k bp, and a total size of 32.1 Mb. In a second step, we error-corrected the PacBio sequence reads with pacBioToCA [103] from the WGS-Assembler version 7 [104] using the Illumina sequence reads. The resulting corrected reads were then used to improve and extend the 454/Roche contigs from the first step using the software PBjelly version 12.9.14 [105]. In order to optimize parameters of the assembly tools and to assess the quality of the final assembly we used the CEGMA tool [106] to count the core eukaryotic genes. Higher numbers of complete proteins are an indication of a more complete and accurate assembly.

For *Crithidia expoeki*, we assembled the Pac Bio reads with a local installation of the PacBio SMRT Portal using the 'RS_HGAP_ Assembly.2' assembly protocol after filtering subreads to a minimum length of 500 bp, minimum quality of 0.75, and a seed read length of 8,000bp. A total of 367,242 reads with mean length 7,446 bp remained after filtering. We then used the Celera Assembler using the following settings: genome size = 35 Mb, target coverage = 25, overlapper error rate = 0.07, overlapper min length = 50, overlapper k-mer = 16. Finally, we used Quiver [107] in the polishing step using only the unambiguously mapped reads. We manually inspected the assembly and removed 73 scaffolds with less than 10x coverage.

In order to assess the completeness of the assembly we ran BUSCO v2.0.1 with the protist ensemble database downloaded from the BUSCO website. The option “—long” was set to turn on the Augustus optimization mode. BUSCO was run on the final genome assemblies of *C*. *bombi* and *C*. *expoeki* and for comparison also on the genome of *L*. *major*.

All reads are deposited in the European Nucleotide Archive (ENA) under accession numbers PRJEB21108 (*C*. *bombi*) and PRJEB21109 (*C*. *expoeki*).

### Transcriptome sequencing and assembly

We isolated RNA from the clones of both *Crithidia* species using the RNEasy Mini Kit (Qiagen cat. no 74104) according to manufacturer's instructions. We then sent the extracted RNA to BGI (Beijing Genomics Institute) for sequencing on the Illumina platform, resulting in 53,695,762 paired-end sequencing reads of 100 bp length and 300 bp insert size. We removed read duplicates with filterPCRdupl.pl (condetri: PCRdupl_v1.01.pl) and trimmed adapters with cutadapt [101]. Finally, we used condetri.pl [102]to quality-filter and trim the reads using the default settings, except for parameters lq (set at 15), lfrac (set at 0.05), ml (set at 1) and minlen (set at 40). We assembled the resulting reads into transcripts with the software Trinity (r2012-05-18 [108]) using 'path reinforcement distance' set at 45 and 'group pairs distance' at 600.

### Gene prediction and annotation

We produced automated gene predictions and structural annotations using MAKER2 [109] with the gene prediction tools SNAP [110], Augustus [111], and GeneMark-ES [112]. A *de novo* repeat library was constructed using RepeatModeler version 1.0.5 (http://www.repeatmasker.org/RepeatModeler.htmlh). We combined the proteins from the UniProt Swiss-Prot protein database [113] and all RefSeq proteins of *Leishmania major*, *L*. *mexicana*, *Trypanosoma brucei*, and *T*. *cruzi* available at the National Center for Biotechnology Information (NCBI) as evidence for protein homology. As EST evidence, we used the Trinity assembled transcripts. Two iterative MAKER2-runs were made to produce a final set of gene predictions and structural annotations. In a first run, MAKER2 was set to use EST evidence for predicting gene models (option: *est2genome* = 1) but not use the gene prediction tools. The resulting gene models (gff and fasta files) were collected. The corresponding protein translations where searched against a protein database containing all Swiss-Prot proteins and all RefSeq proteins of *Leishmania major*, *L*. *mexicana*, *Trypanosoma brucei*, and *T*. *cruzi* with BLAST+ v2.2.23 [114] using the algorithm blastp (numbering 8,316 hits at the time). Proteins with an E-value smaller 1 x 10^−8^ and a query and target sequence coverage of at least 50% were collected. 500 of these proteins were randomly selected and the corresponding genes used as input for training the Augustus gene prediction tool according to the Augustus training tutorial (http://bioinf.uni-greifswald.de/augustus/binaries/tutorial/training.html) provided by the Augustus authors. Briefly, an initial training was run with the *etraining* command, then the gene models were optimized with the optimize_augustus.pl script and finally another *etraining* command was run.

The same 500 genes were used to train SNAP according to the tool’s authors workflow. Briefly, the gff-file was converted with *maker2zff* (a MAKER2 accessory script), then the 500 genes were categorized and exported with the *fathom* command and model parameters estimated with *forge*. Finally, new hmm models were created with the *hmm-assembler*.*pl* script. The self-training gene predictor GeneMark-ES was run with the default settings on the genomic sequences, producing a GeneMark hmm-file. The resulting Augustus, SNAP and GeneMark-ES gene models were now used in a second iteration of MAKER2, this time with the option *est2genome* set to 0.

To annotate our predicted genes, we deployed reciprocal best hit BLAST using the protein predictions derived from the MAKER2 analysis and all RefSeq proteins of *Leishmania major* (numbering 8,316 hits at the time) downloaded from NCBI, using custom Perl scripts and BLAST+ v2.2.23 [114] with a cut-off of E ≤ 1 x 10^−8^. We searched for *L*. *major* homologs to all MAKER2-derived proteins and *vice versa*. We inferred further functional annotations from gene ontology terms using Blast2GO version 2.7.0 [61], which used the results of BLAST searches against the nt-database with BLAST+ v2.2.23, using a E-value threshold of of E ≤ 1 x 10^−8^. We ran BLAST locally and imported these results into Blast2GO. We supplemented the BLAST annotations with results from Interproscan, version 5RC7, which we also run locally and imported these results in Blast2GO. The mapping, annotation augmentation (ANNEX) and analysis steps were then performed using the graphical user interface of Blast2GO using the default settings. The resulting annotations were exported as text files. We assessed the over-representation of gene ontology terms with topGO [115]. The lengths of polycistronic gene clusters containing at least two genes and their gene numbers was extracted from the MAKER2-created annotation files using custom Perl scripts.

### Orthologs and synteny

To find protein orthologs in the two *Crithidia* genomes to other sequenced trypansosomatids, we used a standalone version of the Orthologous Matrix (OMA.1.0.5) [116,117]. For the comparative analyses we used eight taxa. In addition to the annotated proteins of *C*. *bombi* and *C*. *expoeki* (this study), these were the protein libraries (annotated proteins) of the following taxa, generously made available in the TriTryp data base [118]: *Crithidia fasciculata* (TriTrypDB-33_CfasciculataCfCl_AnnotatedProteins; provider: BeverleyLab, by permission), *Leptomonas pyrrhocoris* (TriTrypDB-29_LpyrrhocorisH10_AnnotatedProteins; provider: LukesLab), *Leptomonas seymouri* (TriTrypDB33_LseymouriATCC30220_AnnotatedProteins; provider: YurchenkoLab), *Blechomonas ayalai* (TriTrypDB-33_BayalaiB08-376_AnnotatedProteins; provider: YurchenkoLab) *Leishmania major* Friedlin (TriTrypDB-29_LmajorFriedlin_AnnotateProteins provider: GeneDB), and *Trypanosoma brucei* (TriTrypDB-29_TbruceiTREU927_AnnotatedProteins provider: GeneDB). The choice was guided by having representatives of closer and more distant taxa within the Trypanosomatidae [20,119], and was limited by the computing time needed to run the the identification of orthologs with OMA, and the analysis of signatures of selection, respectively. To confirm the validity of orthologs, we used reciprocal best hit BLAST searches. In our comparative study, we could not include *Crithidia mellificae*/*Lotmaria passim* ([17,47], as the protein database is not available.

Synteny analysis was done with SyMap, version 4.2 [59]. The genome assembly of each species was loaded into SyMap using a minimum contig length threshold of 2000 bp. Alignment and synteny analysis of *C*. *bombi* and *C*. *expoeki* versus all other species was done using the default parameters, with the exception of activating the "merge blocks" option.

### Phylogenetics

We *a priori* selected a number of interesting genes—based on the biology of trypanosomatid genes discussed in the literature and as indicated for each case below—and extracted the protein sequences for both species of *Crithidia*, based on orthology to *L*. *major*. For the phylogenetic reconstruction of gene trees, we started with the orthologs identified by OMA, within the set of eight species, as defined above. After identification of all orthologous sequences in this set, we extracted all genes that carried a particular annotation (such as 'gp63', or 'amastin') in at least one of the annotations of the genes listed in the OMA groups. Typically, this was found to be the annotations for *L*. *major*, arguably the best characterized genome in the set. After identification of all orthologous sequences, we first checked for outliers with a simple neighbour-joining tree of the aligned sequences. Outliers, if any, were checked by submitting the protein sequence to HMMSCAN (biosequence analysis using profile hidden Markov models; available web service: https://www.ebi.ac.uk/Tools/hmmer/search/phmmer). Outliers were removed if the so identified domains were of doubtful support (eventually, only a few cases were removed). Final alignment was subsequently done with MAFFT as web tool (http://www.ebi.ac.uk/Tools/msa/mafft), and the aligned sequences submitted to MrBayes [120] (v3.2.6s) to produce phylogenetic trees, with default settings of four chains, two random start trees, a burn-in period of 25% of trees, and a total of, typically, 10 Mio generations. We used the consensus tree for further visualisation in FigTree v.1.4.2. [70].

### Signatures of selection

We analyzed the signatures of selection and estimated rates of evolution using eight taxa, resulting in tests of 2,934 one-to-one orthology groups, i.e. the set containing a sequence for each of the taxa, as identified by OMA. Alignments were done with prank, and either left un-trimmed, or trimmed with Gblocks with two trimming options, either the stringent ('strict trimming') or relaxed criteria ('relaxed trimming') [121]. We subsequently used the PAML v4.9 package [122], which implements likelihood-based codon models, for calculating the respective statistics. We identified the best model using likelihood ratios for the best fitting model among pairs of nested models. These differed solely in *ω*, the ratio of non-synonymous to synonymous substitutions (*ω* = *dN/dS*). We took *ω >* 1 to indicate positive (diversifying) selection, while *ω <* 1 and *ω* = 1 indicates negative (purifying) and neutral selection, respectively. Functional and structural constraints mean that most sites in functional genes are conserved, hence, the average *ω* is not a good indicator for positive selection [123,124]. We instead used the M7- and M8-models from PAML to test for the presence of positively selected sites [123]. In both models, can vary from site-to-site, based on a Beta-distribution in the interval (0, 1), divided into 11 discrete categories, with the last category (*ω*10) allowed to be ≥ 1. The model also calculates the proportion of sites, *p*, associated with *ω*10, i.e. the proportion of genes under positive selection, which is also the average *dN/dS*-ratio for those sites.

We used the branch-site model (BS) [125,126] to identify episodes of positive selection on the connecting branches between clades. In our case, we *a priori* assigned the branch to the genus *Crithidia* to the foreground, which allows testing for selection on the branch connecting *Crithidia* to the other species, such that foreground sites are constrained, whilst background branches are either constrained or evolving neutrally. We then compared the branch-site model for each orthology group to its corresponding null model, which assumes no difference between foreground and background branches, to identify orthology groups where the branch-site model better describes the evolution of these genes than the null model. In the BS-model, *ω* is divided into three categories with (0 *< ω <* 1), neutral (*ω* = 1), and positively selected (*ω*2 *>* 1; constrained to foreground branches); it returns the proportion of sites, *p*, associated with *ω*2, i.e. the proportion of genes under positive selection. We used three different initial estimates for *ω* and initialized branch lengths to values derived from maximum-likelihood trees constructed in PhyML [127] (using the LG substitution matrix, optimizing only branch-lengths, since the correct topology is known), such that PAML was not caught in local optima. Multiple testing was accounted for with the method of Benjamini-Hochberg [128].

## Supporting information

S1 Fig Synteny of *C*. *bombi* with other taxa. Synteny graph between *C*. *bombi* and other genomes created with Symap 4.2 [59,60]. The plot shows all syntenic blocks between the scaffolds of *C*. *bombi* (upper half of the circle) mapping to scaffolds of the other species in the set (bottom half of the circle; species indicated below). Each coloured block indicates a scaffold of the respective genome. Syntenic blocks are linked with lines in the colour of the *C*. *bombi* scaffold.
(TIF)

S2 Fig Synteny of *C*. *expoeki* with other taxa. Synteny graph between *C*. *expoeki* and other genomes created with Symap 4.2. For further information, see legend to S1 Fig.
(TIF)

S3 Fig Orthologs of *C*. *bomsbi* and *C*. *expoeki* in relation to other taxa. Shown are unrooted trees visualized with FigTree v.1.4.2 [70]; sequences from *C*. *bombi* (in red), and *C*. *expoeki* (in orange) shown in colour for clarity. Sequences of *Bodo saltans* (Kinetoplastida, Bodonidae; in bold black) represent a distant, outgroup kinetoplastid. Sequence labels as in TriTryp data base, and as named here for the two species under study. Branch values are posterior probabilities (PP), only values of PP < 1 show here, all other cases have reported PP = 1. **(a) *Tryparedoxin***. A total of 96 aligned, orthologous sequences were subjected to MrBayes (default settings, with 10 Mio generations and 25% burn-in fraction; convergence: S.D. of split frequencies = 0.01) to construct the consensus tree shown here. **(b) *RAD51***. A total of 31 aligned, orthologous sequences were subjected to MrBayes (default settings, with 10 Mio generations and 25% burn-in fraction; convergence: S.D. of split frequencies = 0.005) to construct the consensus tree shown here.
(TIF)

S4 Fig M8-model. Number of orthologous groups (among 8 taxa) that tested significant for positive selection across the whole phylogeny (M8 *vs*. M7 model). 380 groups were common to all trimming strategies used in Gblocks (strategies were 'none', 'relaxed', 'strict'). Compare S5 File (M8 vs M7 model).
(TIF)

S5 Fig BS-model. Number of orthologous groups (among 8 taxa) that tested significant for positive selection on the branch leading to *Crithidia* (BS-model). 86 groups were common to all trimming strategies used in Gblocks (strategies were 'none', 'relaxed', 'strict'). Compare S6 File (BS model).
(TIF)

S6 Fig ALG-genes. (a) The canonical pathway for the synthesis of glycan by way of additions catalysed by ALG glycosyltransferases. (b) Alignment of genes involved in N-glycan precursor synthesis to scaffolds in the *C*. *bombi* genome.
(TIF)

S7 Fig STT3. Alignment of Stt3 proteins from yeast and kinetoplastids to a scaffold in the *C*. *bombi* genome (scaffold 3/64).
(TIF)

S8 Fig Functional test of *SST3*-substitutes. *C*. *bombi*-derived *CbSTT3A*, but presumably not *CbSTT3B*, can complement the defective mutant *stt3Δ* from yeast (*Saccharomyces cerevisiae*) as shown by the appearance of a product. The background was *stt3Δ*, harbouring two plasmids, expressing *C*. *bombi*-derived *STT3* (*LEU2* marker) and yeast *STT3* (*URA3* marker); incubation at 30° C and 4 days. The conditions were with and without 5-FOA (5-fluoroorotic acid), which, in yeast genetics, is used to select for the absence of the *URA3*-plasmids.
(TIFF)

S1 Table Distribution of exons. (DOCX)

S2 Table Statistics of synteny. (DOCX)

S3 Table *gp63* orthologs (fasta file) (text format). (TXT)

S4 Table Libraries. (DOCX)

S1 File Crithidia-bombi.GDC.2013.v1.gff. The file contains the gene list for *C*. *bombi* (text format).
(TXT)

S2 File Crithidia-expoeki.GDC.2015.v1.gff. The file contains the gene list for *C*. *expoeki* (text format).
(TXT)

S3 File Pairwise orthologs. List of pairwise orthologs found by OMA. Each species pair in a separate sheet. Legend in first sheet (Excel.xlsx format).
(XLSX)

S4 File SignificantGroups. Sheet 'M8.strict.sig': significant groups identified with the M8-model under strict trimming; sheet 'BS.strict.sig': the same for the BS-model. Legend in first sheet. Entries are the Group IDs (number assigned by OMA) of orthologous groups tested for evidence of positive selection. Within a group, gene names assigned in *C*. *bombi*, *C*. *expoeki* as defiend in this study, and for the other taxa as defined in TriTrypDB. Only groups testing positively after BH-correction included here. Functional annotations for the genes in an orthologous group refer to *C*. *bombi*-annotations, and are given by a Hit Description, the GO-term, and a general function term (Excel.xlsx format).
(XLSX)

S5 File M8 model. Entries are the Group IDs (number assigned by OMA) of orthologous group tested for evidence of positive selection after BH-correction. The three sheets refer to different trimmings strategies (none, relaxed, strict) when using Gblocks (Excel.xlsx format). Legend in first sheet.
(XLSX)

S6 File BS model. Entries are the Entries are the Group IDs (number assigned by OMA) of orthologous group tested for evidence of positive selection after BH-correction. Sho The three sheets refer to different trimmings strategies (none, relaxed, strict) when using Gblocks (Excel.xlsx format). Legend in first sheet.
(XLSX)
