# Supplementary figures and images for: The genomes of Crithidia bombi and C. expoeki, common parasites of bumblebees
Source: PLoS One. 2018 Jan 5;13(1):e0189738. doi: 10.1371/journal.pone.0189738 (PMC5755769; doi:10.1371/journal.pone.0189738)

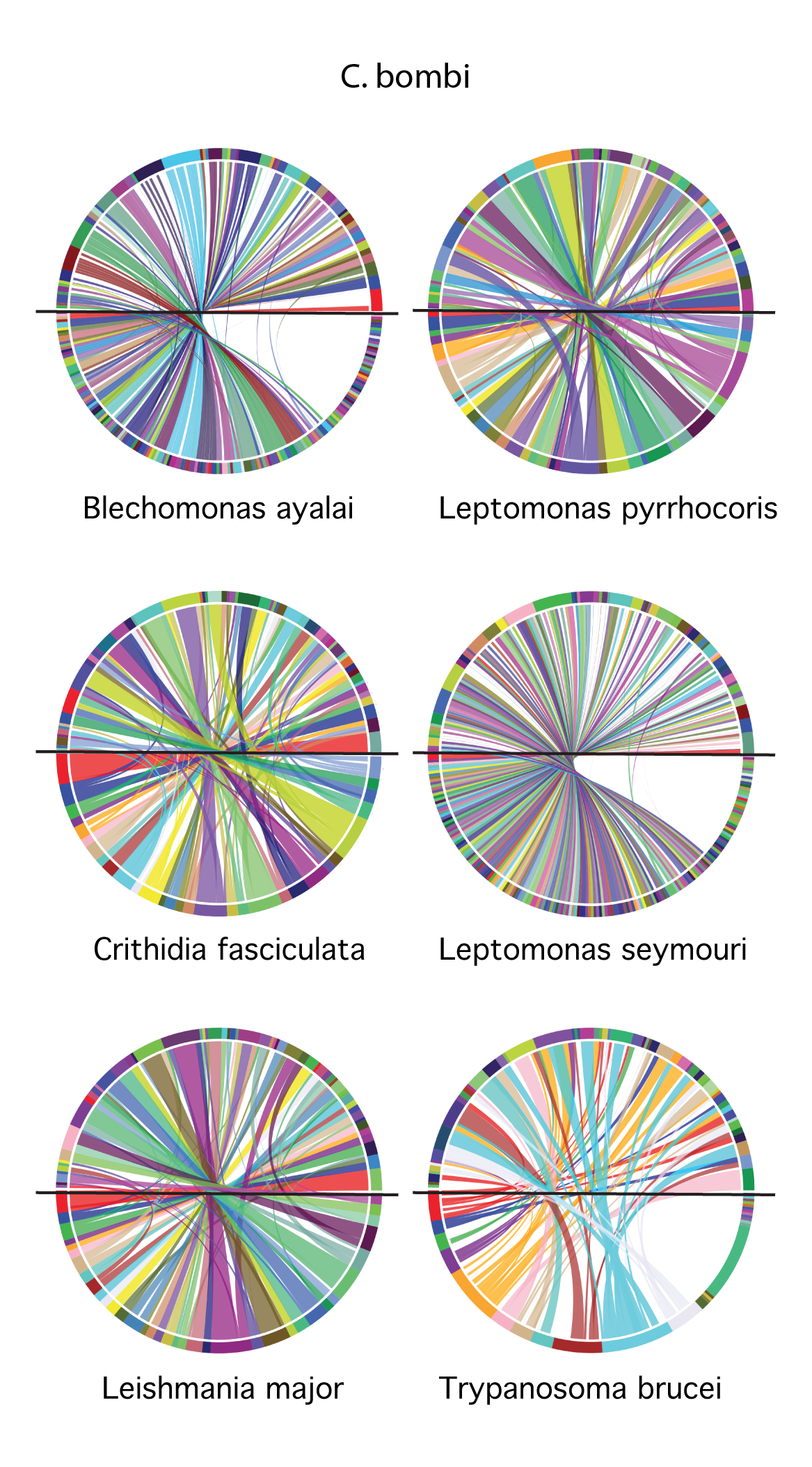

Supplement: S1 Fig — Synteny graph between C. bombi and other genomes created with Symap 4.2 [59,60]. The plot shows all syntenic blocks between the scaffolds of C. bombi (upper half of the circle) mapping to scaffolds of the other species in the set (bottom half of the circle; species indicated below). Each coloured block indicates a scaffold of the respective genome. Syntenic blocks are linked with lines in the colour of the C. bombi scaffold. (TIF) [file pone.0189738.s001.tif]

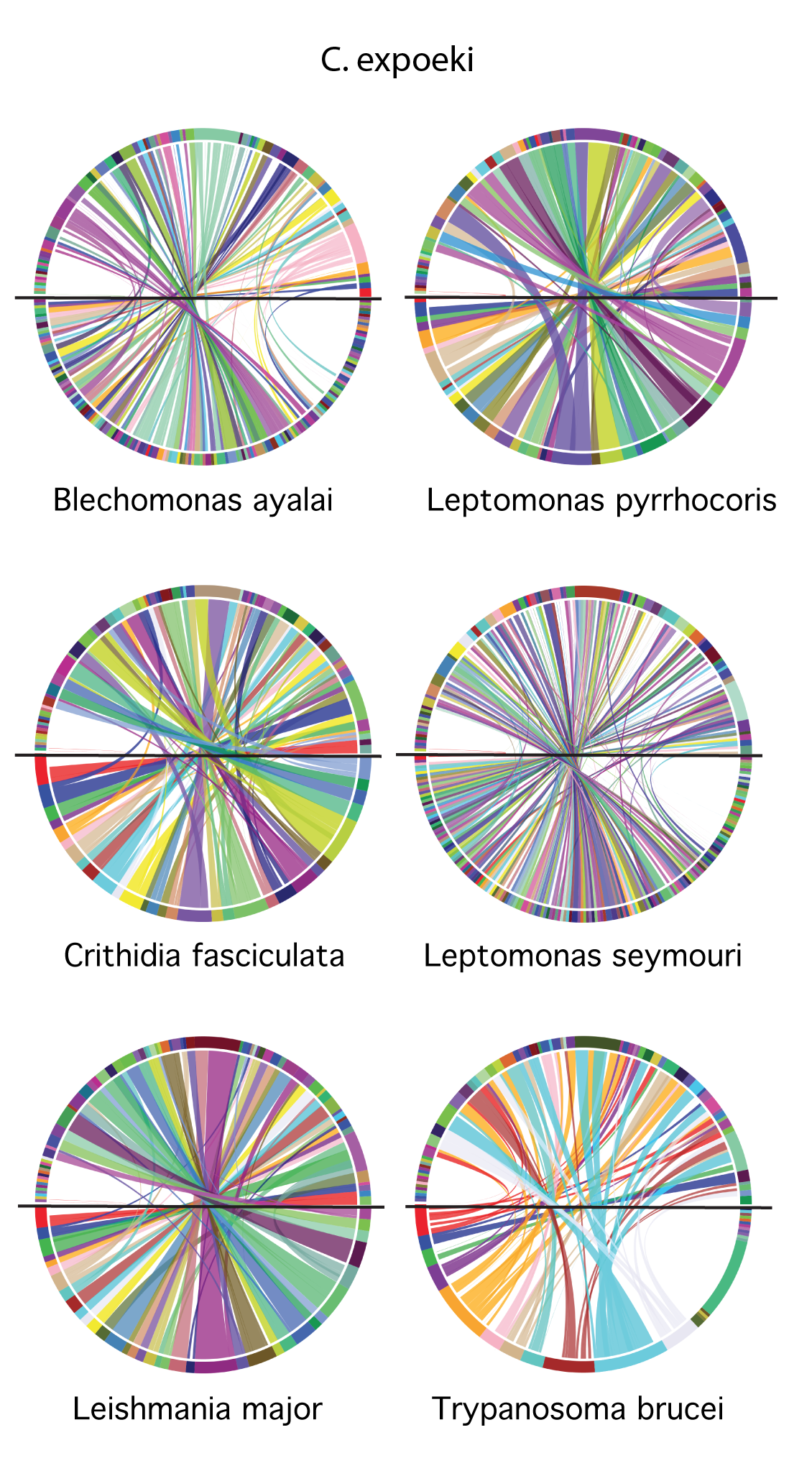

Supplement: S2 Fig — Synteny graph between C. expoeki and other genomes created with Symap 4.2. For further information, see legend to S1 Fig. (TIF) [file pone.0189738.s002.tif]

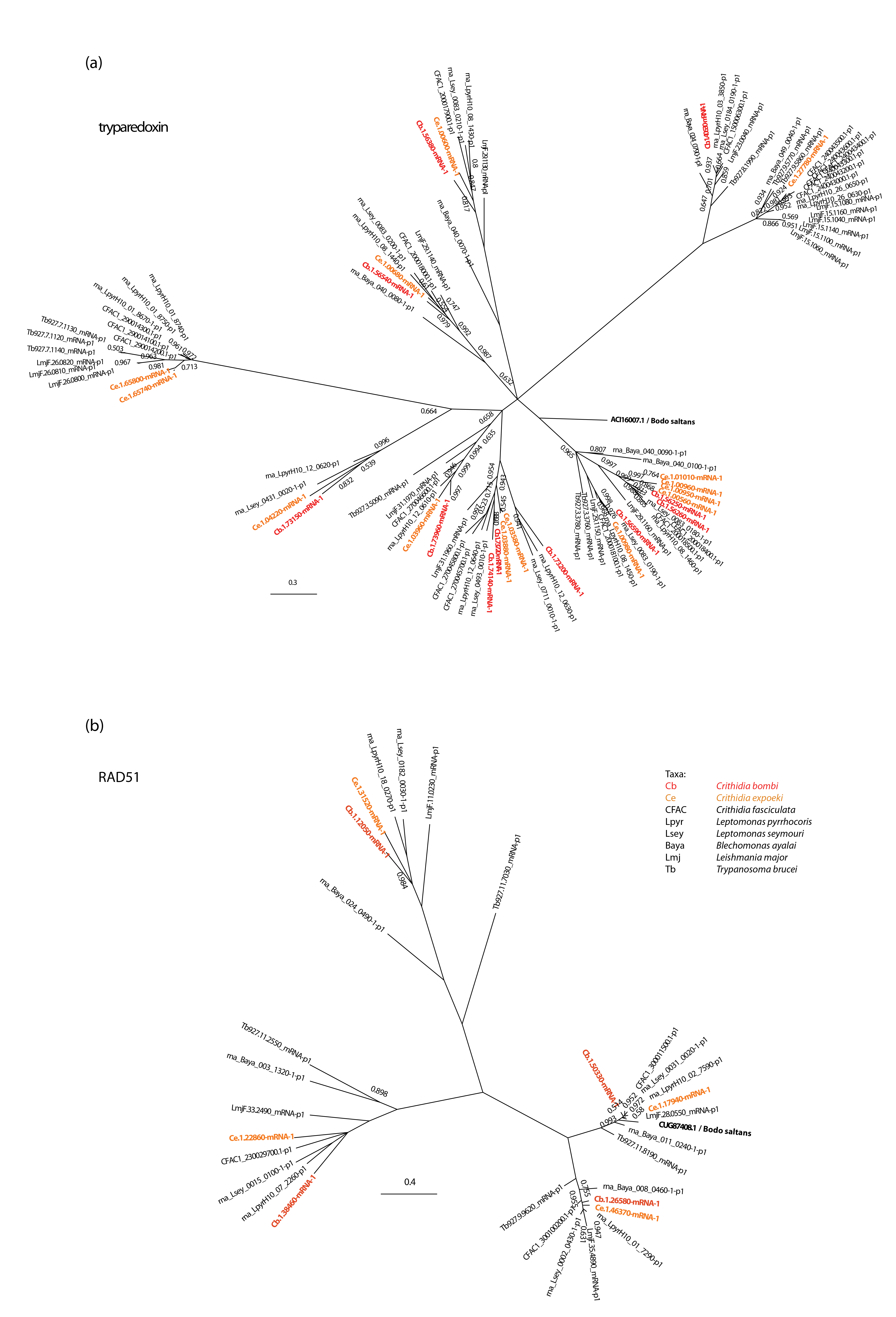

Supplement: S3 Fig — Shown are unrooted trees visualized with FigTree v.1.4.2 [70]; sequences from C. bombi (in red), and C. expoeki (in orange) shown in colour for clarity. Sequences of Bodo saltans (Kinetoplastida, Bodonidae; in bold black) represent a distant, outgroup kinetoplastid. Sequence labels as in TriTryp data base, and as named here for the two species under study. Branch values are posterior probabilities (PP), only values of PP < 1 show here, all other cases have reported PP = 1. (a) Tryparedoxin. A total of 96 aligned, orthologous sequences were subjected to MrBayes (default settings, with 10 Mio generations and 25% burn-in fraction; convergence: S.D. of split frequencies = 0.01) to construct the consensus tree shown here. (b) RAD51. A total of 31 aligned, orthologous sequences were subjected to MrBayes (default settings, with 10 Mio generations and 25% burn-in fraction; convergence: S.D. of split frequencies = 0.005) to construct the consensus tree shown here. (TIF) [file pone.0189738.s003.tif]

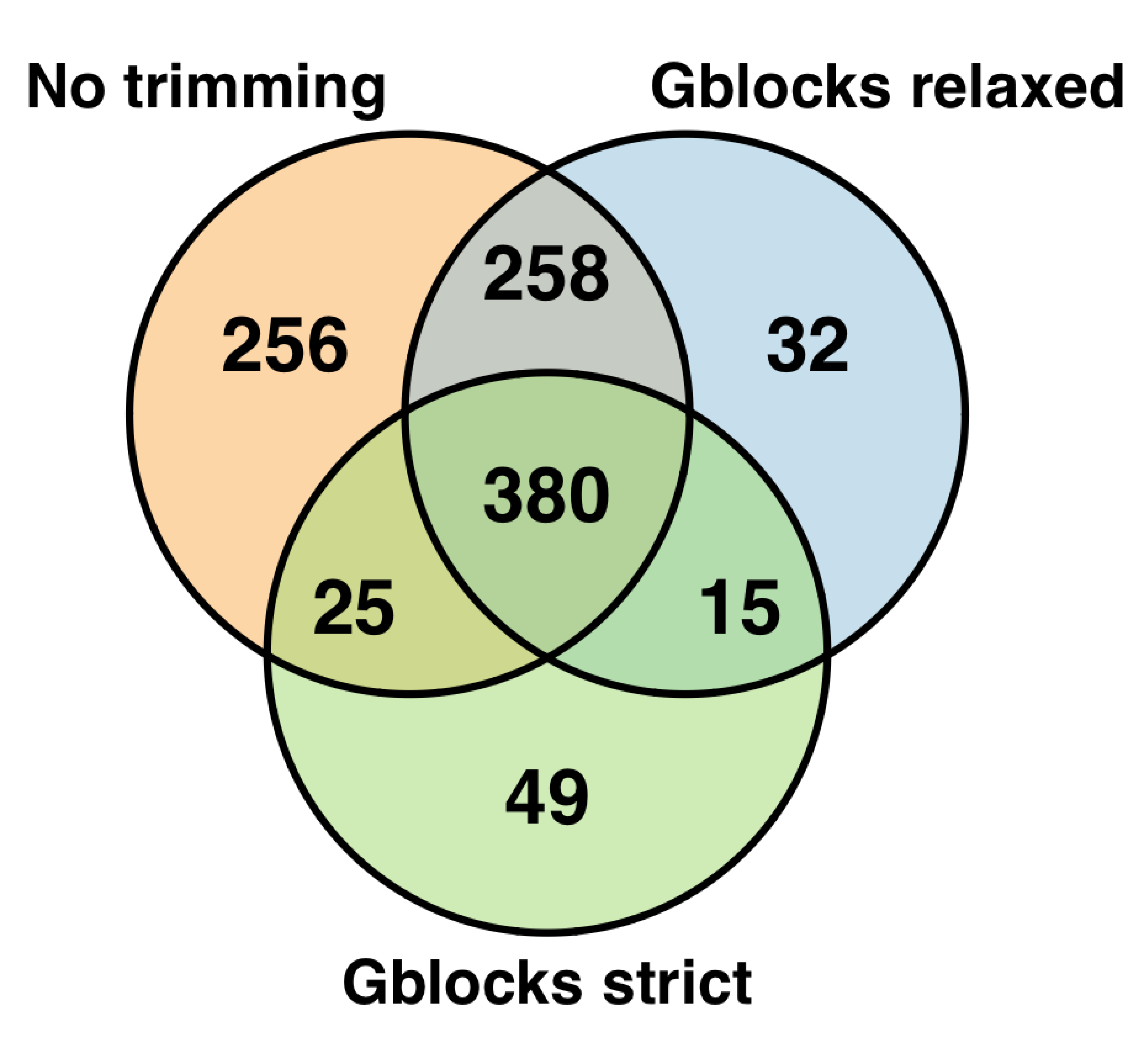

Supplement: S4 Fig — Number of orthologous groups (among 8 taxa) that tested significant for positive selection across the whole phylogeny (M8 vs. M7 model). 380 groups were common to all trimming strategies used in Gblocks (strategies were 'none', 'relaxed', 'strict'). Compare S5 File (M8 vs M7 model). (TIF) [file pone.0189738.s004.tif]

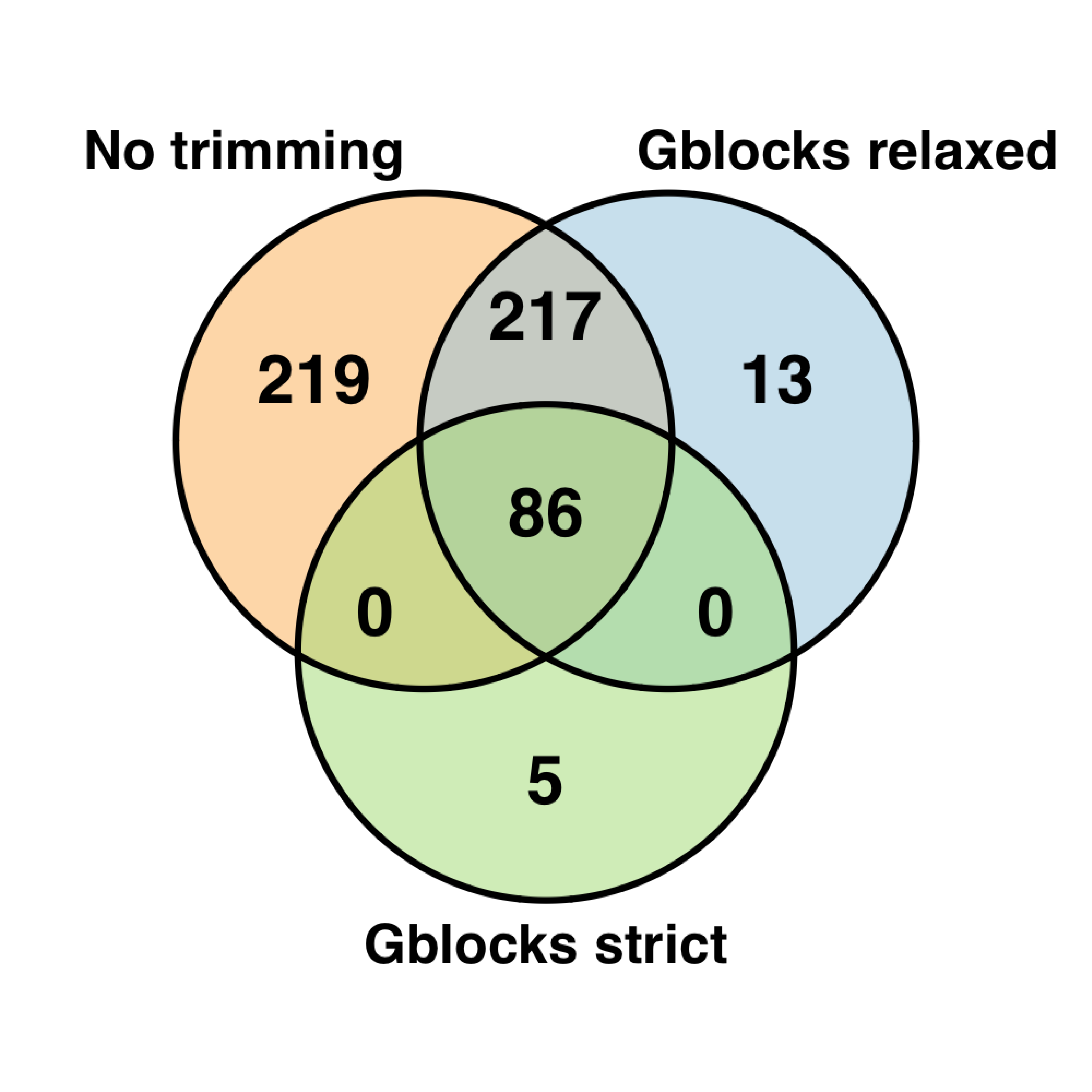

Supplement: S5 Fig — Number of orthologous groups (among 8 taxa) that tested significant for positive selection on the branch leading to Crithidia (BS-model). 86 groups were common to all trimming strategies used in Gblocks (strategies were 'none', 'relaxed', 'strict'). Compare S6 File (BS model). (TIF) [file pone.0189738.s005.tif]

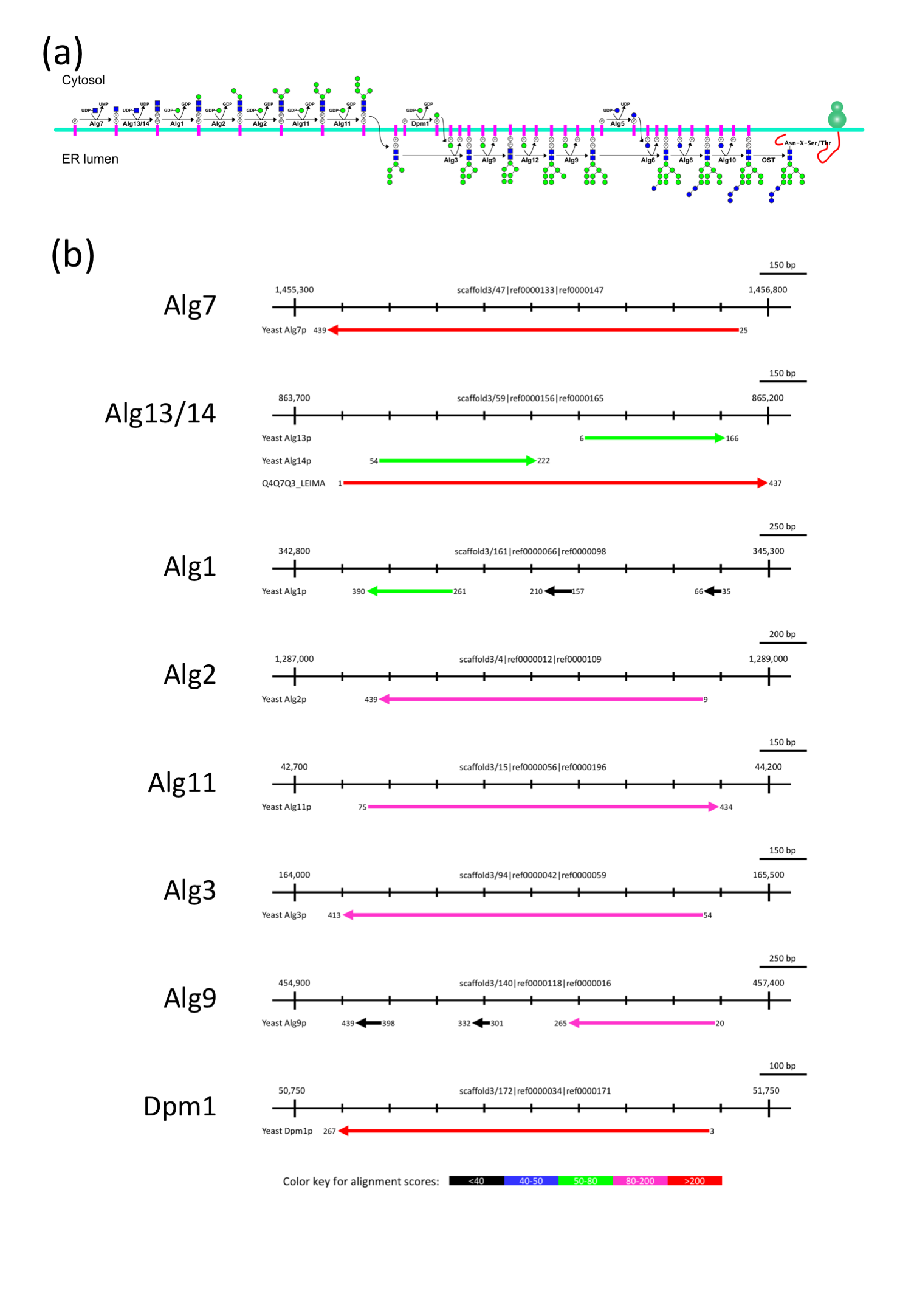

Supplement: S6 Fig — (a) The canonical pathway for the synthesis of glycan by way of additions catalysed by ALG glycosyltransferases. (b) Alignment of genes involved in N-glycan precursor synthesis to scaffolds in the C. bombi genome. (TIF) [file pone.0189738.s006.tif]

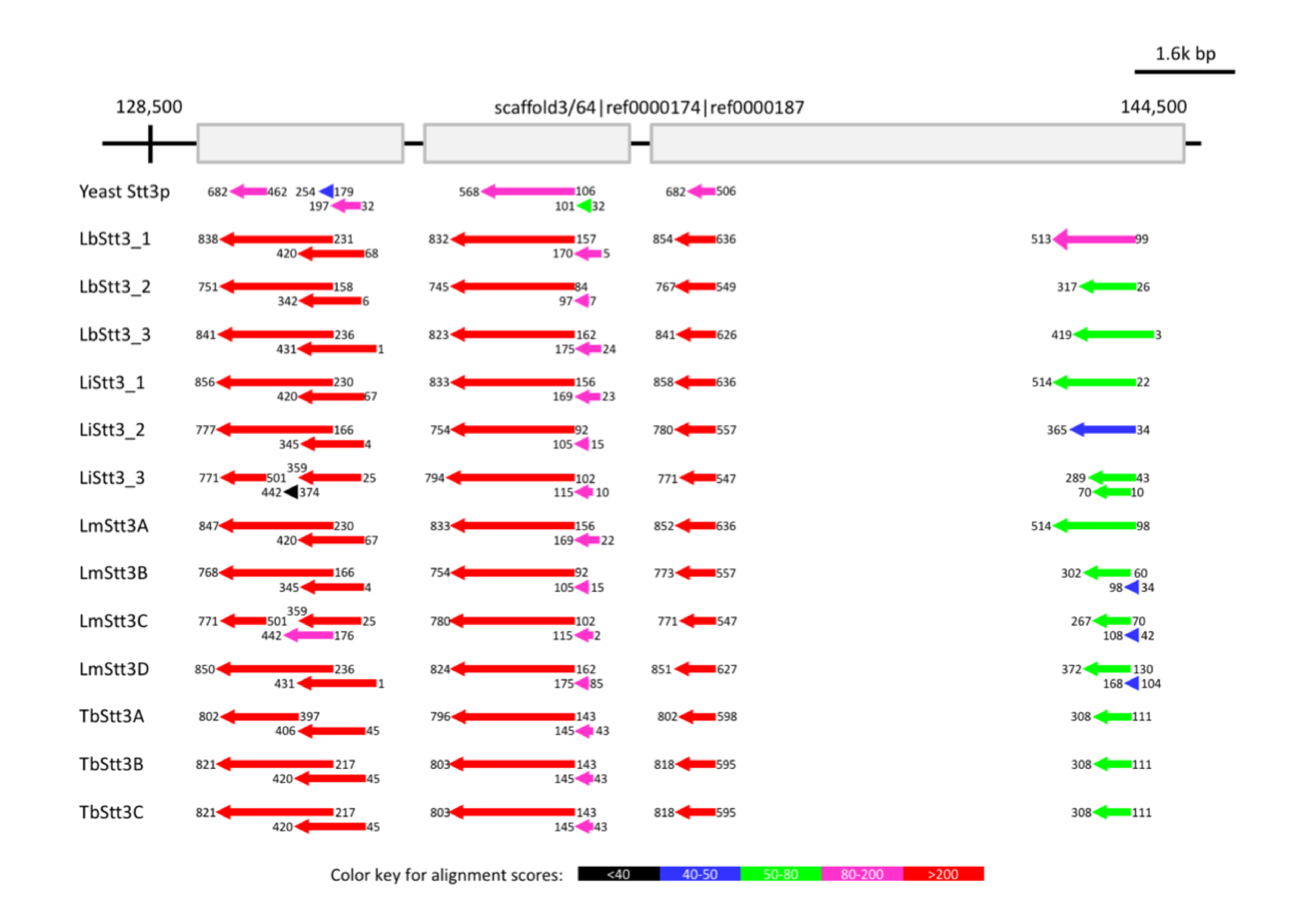

Supplement: S7 Fig — Alignment of Stt3 proteins from yeast and kinetoplastids to a scaffold in the C. bombi genome (scaffold 3/64). (TIF) [file pone.0189738.s007.tif]

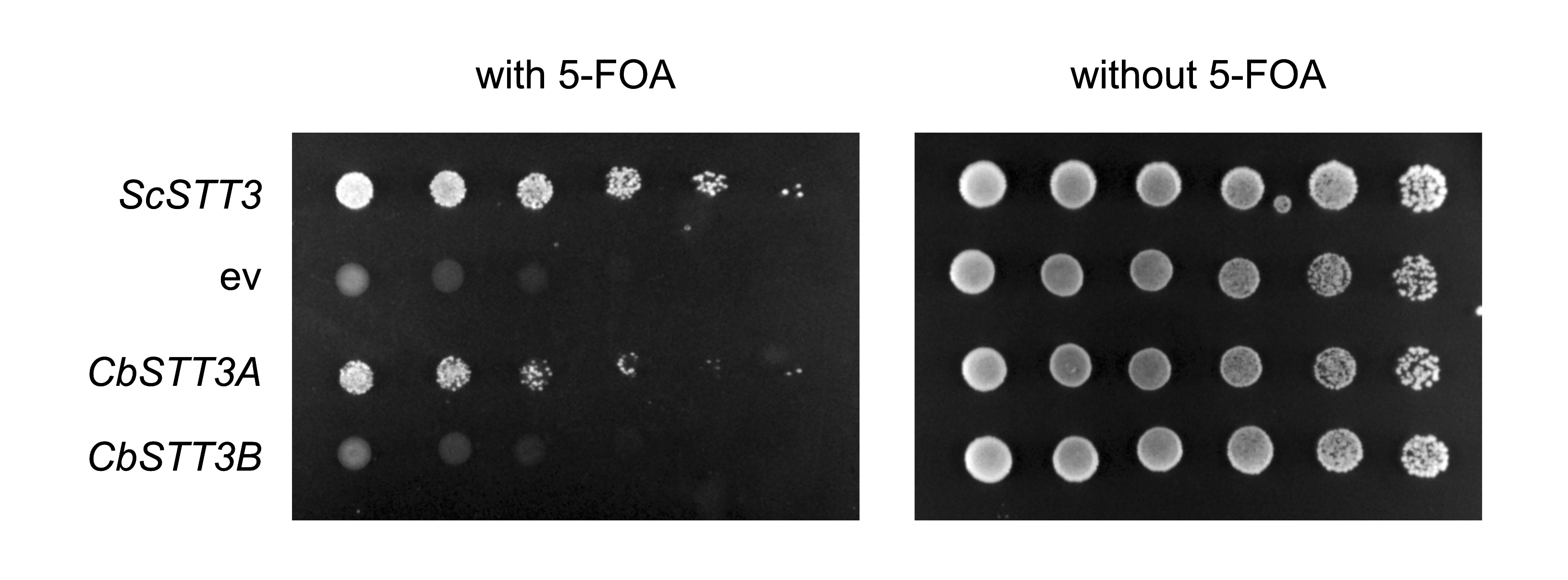

Supplement: S8 Fig — C. bombi-derived CbSTT3A, but presumably not CbSTT3B, can complement the defective mutant stt3Δ from yeast (Saccharomyces cerevisiae) as shown by the appearance of a product. The background was stt3Δ, harbouring two plasmids, expressing C. bombi-derived STT3 (LEU2 marker) and yeast STT3 (URA3 marker); incubation at 30° C and 4 days. The conditions were with and without 5-FOA (5-fluoroorotic acid), which, in yeast genetics, is used to select for the absence of the URA3-plasmids. (TIFF) [file pone.0189738.s008.tiff]
