# Supplementary material for: The genomes of Crithidia bombi and C. expoeki, common parasites of bumblebees
Source: PLoS One. 2018 Jan 5;13(1):e0189738. doi: 10.1371/journal.pone.0189738 (PMC5755769; doi:10.1371/journal.pone.0189738)
Supplement: S1 Table — (DOCX) [file pone.0189738.s009.docx]

**S1 Table. Distribution of exons.**

Number of exons per gene in *C. bombi* and *C. expoeki*

|  | number of genes | |
| --- | --- | --- |
| number  of exons | *C. bombi* | *C. expoeki* |
| 1 | 6,967 | 6,465 |
| 2 | 700 | 928 |
| 3 | 61 | 256 |
| 4 | 33 | 95 |
| 5 | 18 | 46 |
| 6 | 15 | 22 |
| 7 | 7 | 19 |
| 8 | 5 | 9 |
| 9 | 2 | 9 |
| 10 | - | 2 |
| all | 7,808 | 7,851 |
