# Supplementary material for: The genomes of Crithidia bombi and C. expoeki, common parasites of bumblebees
Source: PLoS One. 2018 Jan 5;13(1):e0189738. doi: 10.1371/journal.pone.0189738 (PMC5755769; doi:10.1371/journal.pone.0189738)
Supplement: S2 Table — (DOCX) [file pone.0189738.s010.docx]

**S2 Table. Statistics of synteny.**

Shown are either *C. bombi* or *C. expoeki*, paired with another species from the set (first column), and the respective statistics for each member of the pair. Pairwise synteny statistics computed with SyMap.

|  | | | |  | |  | |  | |  | |  | |
| --- | --- | --- | --- | --- | --- | --- | --- | --- | --- | --- | --- | --- | --- |
| Species pair | Number of blocks | Percent coverage^1^ | Percent double coverage^2^ | | Inverted^3^ | | Blocks <100kb | | Blocks 100kb-1Mb | | Blocks  1Mb-10Mb | |  |
| *C. bombi* | 99 | 91% | 0% | | 15 | | 18 | | 80 | | 1 | |  |
| *C. expoeki* | 99 | 82% | 0% | | 15 | | 18 | | 79 | | 2 | |  |
|  |  |  |  | |  | |  | |  | |  | |  |
| *C. expoeki* | 77 | 73% | 2% | | 36 | | 7 | | 69 | | 1 | |  |
| *T. brucei* | 77 | 49% | 2% | | 36 | | 18 | | 57 | | 2 | |  |
|  |  |  |  | |  | |  | |  | |  | |  |
| *C. expoeki* | 260 | 58% | 0% | | 106 | | 208 | | 52 | | 0 | |  |
| *L. seymouri* | 260 | 68% | 0% | | 106 | | 216 | | 44 | | 0 | |  |
|  |  |  |  | |  | |  | |  | |  | |  |
| *C. expoeki* | 76 | 84% | 0% | | 30 | | 10 | | 63 | | 3 | |  |
| *L. pyrrhocoris* | 76 | 92% | 2% | | 30 | | 9 | | 62 | | 5 | |  |
|  |  |  |  | |  | |  | |  | |  | |  |
| *C. expoeki* | 75 | 84% | 1% | | 33 | | 8 | | 63 | | 4 | |  |
| *L. major* | 75 | 93% | 0% | | 33 | | 7 | | 63 | | 5 | |  |
|  |  |  |  | |  | |  | |  | |  | |  |
| *C. expoeki* | 86 | 85% | 0% | | 33 | | 15 | | 69 | | 2 | |  |
| *C. fasciculata* | 86 | 92% | 1% | | 33 | | 15 | | 67 | | 4 | |  |
|  |  |  |  | |  | |  | |  | |  | |  |
| *C. expoeki* | 153 | 56% | 1% | | 72 | | 75 | | 78 | | 0 | |  |
| *B. ayalai* | 153 | 60% | 0% | | 72 | | 113 | | 40 | | 0 | |  |
|  |  |  |  | |  | |  | |  | |  | |  |
| *C. bombi* | 71 | 79% | 4% | | 29 | | 8 | | 58 | | 5 | |  |
| *T. brucei* | 71 | 47% | 2% | | 29 | | 18 | | 52 | | 1 | |  |
|  |  |  |  | |  | |  | |  | |  | |  |
| *C. bombi* | 280 | 67% | 0% | | 5 | | 225 | | 55 | | 0 | |  |
| *L. seymouri* | 280 | 70% | 0% | | 5 | | 234 | | 46 | | 0 | |  |
|  |  |  |  | |  | |  | |  | |  | |  |
| *C. bombi* | 82 | 94% | 0% | | 23 | | 14 | | 64 | | 4 | |  |
| *L. pyrrhocoris* | 82 | 94% | 0% | | 23 | | 14 | | 64 | | 4 | |  |
|  |  |  |  | |  | |  | |  | |  | |  |
| *C. bombi* | 81 | 94% | 0% | | 20 | | 16 | | 60 | | 5 | |  |
| *L. major* | 81 | 95% | 1% | | 20 | | 15 | | 60 | | 6 | |  |
|  |  |  |  | |  | |  | |  | |  | |  |
| *C. bombi* | 91 | 94% | 1% | | 29 | | 17 | | 69 | | 5 | |  |
| *C. fasciculata* | 91 | 93% | 1% | | 29 | | 16 | | 70 | | 5 | |  |
|  |  |  |  | |  | |  | |  | |  | |  |
| *C. bombi* | 171 | 62% | 0% | | 11 | | 90 | | 81 | | 0 | |  |
| *B. ayalai* | 171 | 61% | 0% | | 11 | | 135 | | 36 | | 0 | |  |

^1^ Percent coverage: Percent of total sequence length covered by synteny blocks.

^2^ Percent double coverage: Percent of total sequence length covered by two or more synteny blocks (i.e., mapping to a putative duplication).

^3^ Inverted: Number of blocks which are inverted.
