# Supplementary material for: The genomes of Crithidia bombi and C. expoeki, common parasites of bumblebees
Source: PLoS One. 2018 Jan 5;13(1):e0189738. doi: 10.1371/journal.pone.0189738 (PMC5755769; doi:10.1371/journal.pone.0189738)
Supplement: S4 Table — (DOCX) [file pone.0189738.s012.docx]

**S4 Table. Libraries.**

Sequencing libraries on the Roche/454 GS FLX platform.

| Library | Library type | Insert size | Number of reads |
| --- | --- | --- | --- |
| G7FQSL201 | PE | 20 kb | 932967 |
| G7FQSL202 | PE | 10 kb | 76059 |
| GP9I81001 | PE | 8 kb | 871976 |
| GP9I81002 | PE | 8 kb | 793880 |
| G3X5M9301 | PE | 4 kb | 402594 |
| G3X5M9302 | PE | 4 kb | 390664 |
| G18QOAC01 | PE | 2 kb | 715527 |
| GY3UCQB01 | PE | 2 kb | 856918 |
| GY3UCQB02 | PE | 2 kb | 868776 |
| FX2FBNA01 | SE | - | 603853 |
| FX2FBNA02 | SE | - | 614075 |
